# Characterization of Anticancer Drug Protomers Using Electrospray Ionization and Ion Mobility Spectrometry–Mass Spectrometry

**DOI:** 10.1021/jasms.4c00233

**Published:** 2024-09-27

**Authors:** Pallab Basuri, Marc Safferthal, Borislav Kovacevic, Pascal Schorr, Jerome Riedel, Kevin Pagel, Dietrich A. Volmer

**Affiliations:** † Institute of Chemistry, Humboldt-Universität zu Berlin, 12489 Berlin, Germany; ‡ Institute of Chemistry and Biochemistry, Freie Universität Berlin, 14195 Berlin, Germany; § Division of Physical Chemistry, Ruđer Bošković Institute, 10000 Zagreb, Croatia

## Abstract

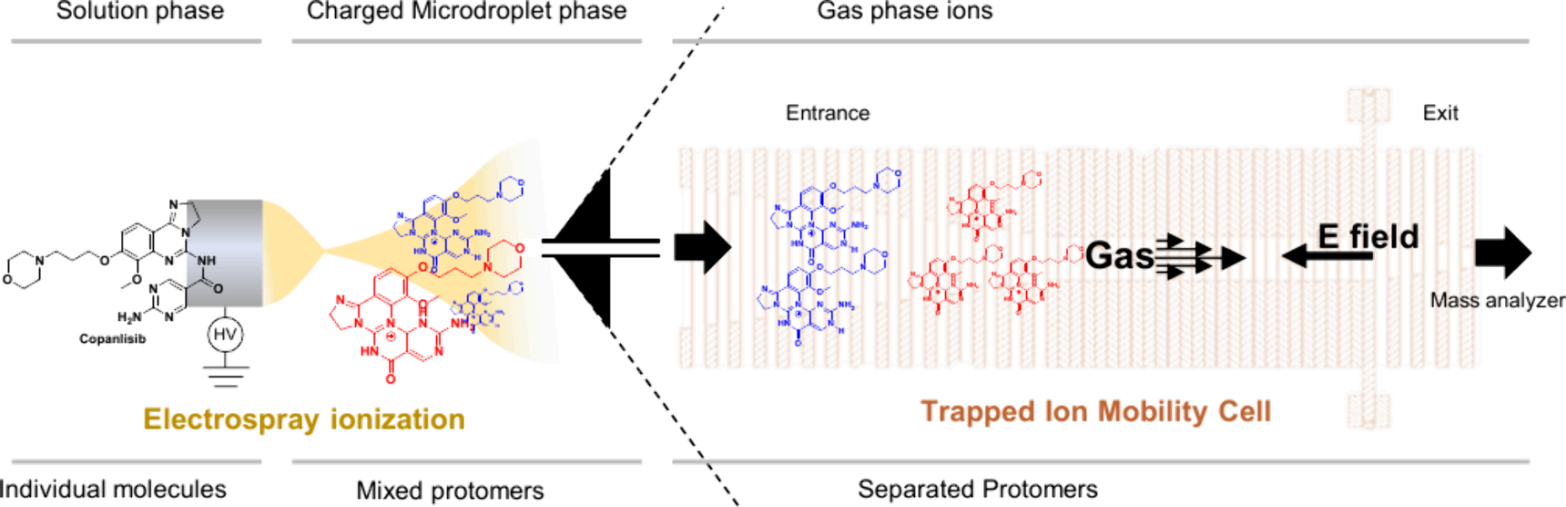

We used electrospray ionization and ion mobility spectrometry–mass spectrometry to detect and characterize the three anticancer drugs palbociclib, copanlisib, and olaparib. Ion mobility–mass spectrometry and density functional theory revealed that these compounds generate isomers during ionization (protomers) due to the presence of multiple protonation sites within their chemical structures. Our work has implications for understanding the solution- and gas-phase chemistry of these molecules during spray-based ionization processes.

## Introduction

Liquid chromatography–tandem mass spectrometry (LC-MS/MS) using electrospray ionization (ESI) has been widely used for screening and quantifying drugs with high sensitivity and selectivity.^1−3^ Unfortunately, developing LC-MS methods is often a tedious process involving sample extraction, pretreatment, preconcentration, and derivatization, which can be time-consuming and laborious.^4,5^ Ambient ionization mass spectrometry techniques such as desorption electrospray ionization,^6^ paper spray ionization,^7^ low-temperature plasma ionization,^8^ and several other variants^9−13^ have greatly helped simplify analysis, as only a little preparation of the sample is required. Nevertheless, there have been reports of analytical variations during quantitative analysis of small molecules in biological samples depending on experimental conditions, including sample preparation.^14−16^ It was shown that this was the result of the formation of multiple isomers (protomers) during ionization via protonation at different sites of the analyte molecules. This can lead to inconsistent quantitative results for multiple reaction monitoring (MRM)-based quantifications using structure-specific product ions if the precursor/product ion ratio depends on the experimental conditions.^15,17^

Identifying protonation and deprotonation sites within a molecule can help determine the extent of protomer formation and the different properties of these isomers.^18^ For example, using traveling wave ion mobility–mass spectrometry (TWIM-MS) with post- and pre-TWIM collision-induced dissociation (CID), Eberlin et al. identified and isolated two coexisting protomers of two isomeric porphyrins.^19^ The protomer resulting from deprotonation at the carboxyl group, which dissociates quickly by CO_2_ loss, was found to make up the majority of the deprotonated porphyrin sampled from a basic methanolic solution. However, a CID-resistant protomer arising from deprotonation at a porphyrine ring was also detected and characterized. Volmer and co-workers illustrated the formation of protomers from several 4-quinolone antibiotics using a combination of ESI, differential ion mobility spectrometry (DMS), CID, and density function theory (DFT).^20^ The individual protomers were readily separated via DMS-MS, and protomer-specific CID spectra from both charge-remote and charge-directed processes were shown for the protomers. The authors demonstrated that solution-phase properties such as pH influence the formation ratio of the isomeric variants generated during ESI. Furthermore, Trevitt et al. showed that ion mobility spectrometry can identify protomers of ciprofloxacin, which results in unique CID mass spectra.^21^ Several other groups also described protomer formation during ionization for many other molecules such as imipramine,^22^*p*-aminobenzoic acid,^23−25^ cyclic peptides,^26^ quinazolines,^27^ opioids,^28^ fentanyls,^29^ and caffeine.^30^

The anticancer drugs investigated in this study contain multiple functional groups within their structures, with potential for the formation of multiple stable protomers if the charge cannot be immediately redistributed within the molecule. Sample preparation, chromatography separation, and mass spectrometry parameters such as types of solvent and buffers, pH, nebulization conditions, droplet desolvation, ionization, and ion optic voltages have the potential to control the number density distribution of these protomers, which will then affect the distribution of fragment ion species in the MS/MS spectra. This can have a strong effect in quantitative and confirmatory analyses, in particular targeted analyses in the MRM mode if these protomers are not preseparated prior to MS analysis, e.g., by ion mobility spectrometry. This was first shown by Kaufmann et al., who observed the erratic behavior of precursor–product ion transition ratios in MRM-based analyses of biological samples after ESI, which strongly depended on the sample matrix the analytes were extracted from.^31^ The authors were able to link this effect to two [M + H]^+^ species of the investigated 4-quinolone drugs from protonation at two sites in the molecule, leading to protomers with entirely different CID spectra. Wang et al. clearly demonstrated that the ratio of two protomers of drug compounds with two basic sites strongly depends on the mobile phase pH, aqueous–organic ratio, and buffer concentration in LC-MS/MS in MRM.^32^

Detection of such isomers for the investigated anticancer drugs in this study will help develop more robust quantitative assays for drug detection in blood or tissues, and it will also provide improved structural information for characterization of the parent drugs and their metabolites.

Here, we used a mass spectrometric method for the characterization of the three anticancer drugs palbociclib, copanlisib, and olaparib, with a particular focus on the characterization of protomers of these molecules using drift tube and trapped ion mobility mass spectrometry as well as density functional theory to better understand the solution- and gas-phase chemistry of these isomeric ions.

## Experimental Section

### Chemicals and Materials

Drug standards were purchased from Sigma-Aldrich (Steinheim, Germany), and aqueous standard solutions were prepared with Milli-Q water and used directly without further treatment.

### Mass Spectrometry

We used a Sciex (Concord, ON, Canada) QTRAP 6500^+^ quadrupole-linear ion trap (QqLIT) MS equipped with a Turbo-V ESI source for measurements of 10 μM aqueous solutions of the drugs. Ion mobility spectrometry (IMS) experiments were performed on a modified Waters (Manchester, UK) Synapt G2-S HDMS Drift Tube (DT) IMS and a Bruker (Bremen, Germany) timsTOF Pro Trapped IMS (TIMS). All IMS experiments were performed in nitrogen drift gas using nano-ESI with nitrogen nebulization. Absolute DT CCS_N2_ values were determined using the stepped-field method.^33,34^ Estimated TIMS CCS_N2_ values were obtained by calibration using an Agilent (Santa Clara, CA, USA) ESI low-concentration tuning mix.

### DFT Calculations

Calculations in the gas phase were performed at the M06-2X/6-311++G(3df,2p)//M06-2X/6-311+G(d,p) level of theory. All structures were optimized without imposing any geometry constraints and were confirmed to represent energy minima on the potential energy surface through the analytical computation of their vibrational frequencies. Gas-phase basicities (GB) were calculated as the negative Gibbs energy Δ*G* of the reaction B + H^+^ → BH^+^ as follows: GB = −{*G*^298^(BH^+^) – [*G*(B^298^) + *G*^298^(H^+^)]}. The Gibbs energy of the proton in the gas phase, *G*^298^(H^+^), has a value of −6.295 kcal·mol^–1^.^35^*G* values of the base and conjugate acid contain the electronic energy *E*_el_ obtained at the M06-2X/6-311++G(3df,2p)//M06-2X/6-311+G(d,p) level of theory and the thermal correction to free energy, *G*_therm_, which sums the zero-point vibrational energy (ZPVE), enthalpic contribution, and entropic contribution at 298 K. Calculation of p*K*_a_ values in water were obtained with the SMD as a continuum solvation model utilizing the same functional and basis set as for gas-phase calculations (see above). All structures were reoptimized in water, with the inclusion of one explicit water molecule in the first solvation shell to account for the contribution to the solvation energy arising from hydrogen bond formation between the solute and solvent. p*K*_a_ values were determined as relative values utilizing an isodesmic reaction approach,^36^ with piperazine (experimental p*K*_a_= 9.73^37^) serving as the reference base. The entire set of calculations was conducted using the Gaussian 16 program package.^38^

The gas-phase geometries of the neutral and protonated species optimized utilizing the M06-2X/6-311+G(d,p) level of theory are included in the Supporting Information (Table S1).

## Results and Discussion

### Protomer Formation

**Scheme 1:**
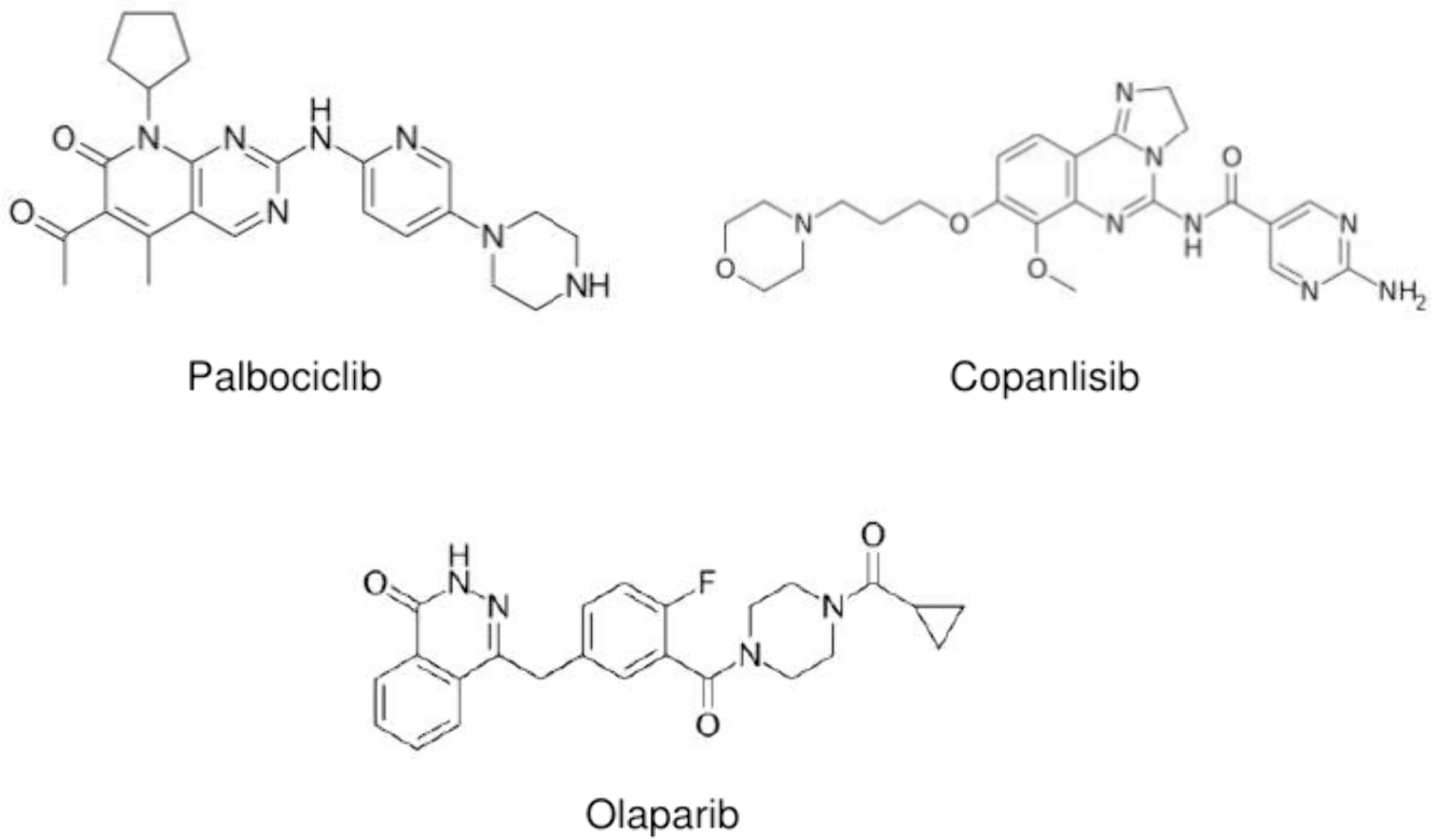
Chemical Structures of the Investigated Anticancer Drugs Palbociclib, Copanlisib and Olaparib

We initially performed ESI-MS of standard aqueous samples of the three investigated drugs (Scheme 1) using a quadrupole-linear ion trap MS instrument. Interestingly, we found doubly protonated species in the full scan mass spectra of palbociclib and copanlisib, which were confirmed by MS/MS experiments. These doubly charged species hint at the existence of two protonation sites at different positions. However, olaparib did not show doubly charged species in the mass spectrum (ESI-MS and MS/MS spectra for the three drugs are summarized in Figures S1–3 in the Supporting Information).

**Figure 1:**
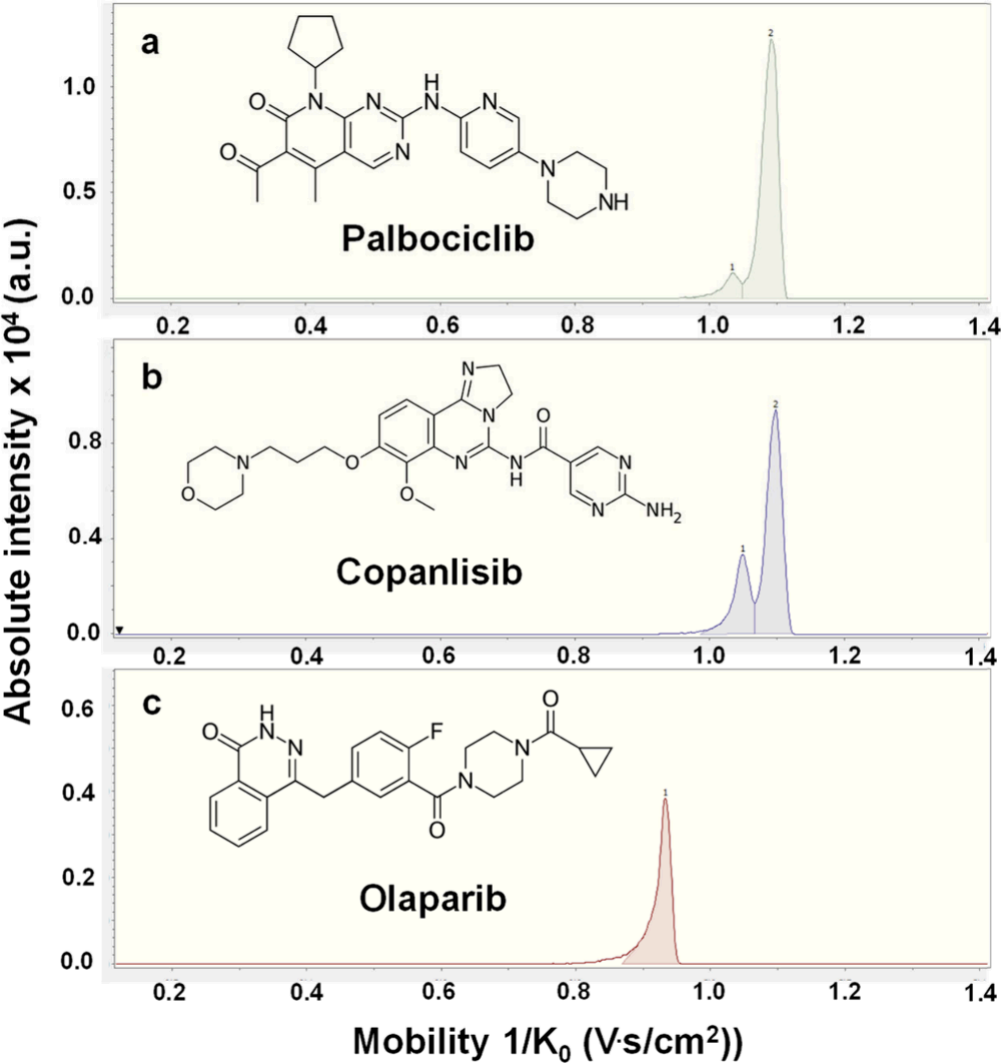
Trapped ion mobility spectra of the eletrosprayed [M + H]^+^ ions of (a) palbociclib, (b) copanlisib, and (c) olaparib acquired using TIMS.

We then conducted ion mobility spectrometry (IMS)-MS experiments to reveal potential protomers from protonation at different positions of the drug molecules during ionization prior to MS analysis. First, we recorded drift tube (DT) IMS separations for the three drugs. While palbociclib and olaparib exhibited only a single peak in the DTIMS separations, copanlisib displayed a minor shoulder next to the major peak at 9.90 ms drift time (the IMS separation is shown in the Supporting Information, Figure S4). The measured experimental CCS values for palbociclib and olaparib were 226 and 193 Å^2^, respectively, whereas for the two peaks of copanlisib in IMS the experimental CCS values were 221 and 228 Å^2^. We observed that the protomers were not well separated in DTIMS due to the low resolving power of the instrument. By means of TIMS, however, we were able to separate two isomers of palbociclib and copanlisib, as shown in Figure 1. Experimental CCS values for the two separated isomers of palbociclib using TIMS-MS were 214 and 226 Å^2^ (Figure 1a). Similarly, the experimental CCS values for copanlisib were 217 and 227 Å^2^ (Figure 1b). We observed a small tailing toward lower mobility values in the olaparib spectrum (Figure 1c). However, no separation of the isomers was observed. We therefore postulate that for olaparib one protomer was formed predominantly during ionization. The experimental CCS value for olaparib was 196 Å^2^. Note that this small tailing in the IMS mobilograms is potentially due to space-charge artifacts from overfilling the mobility analyzer.

**Scheme 2:**
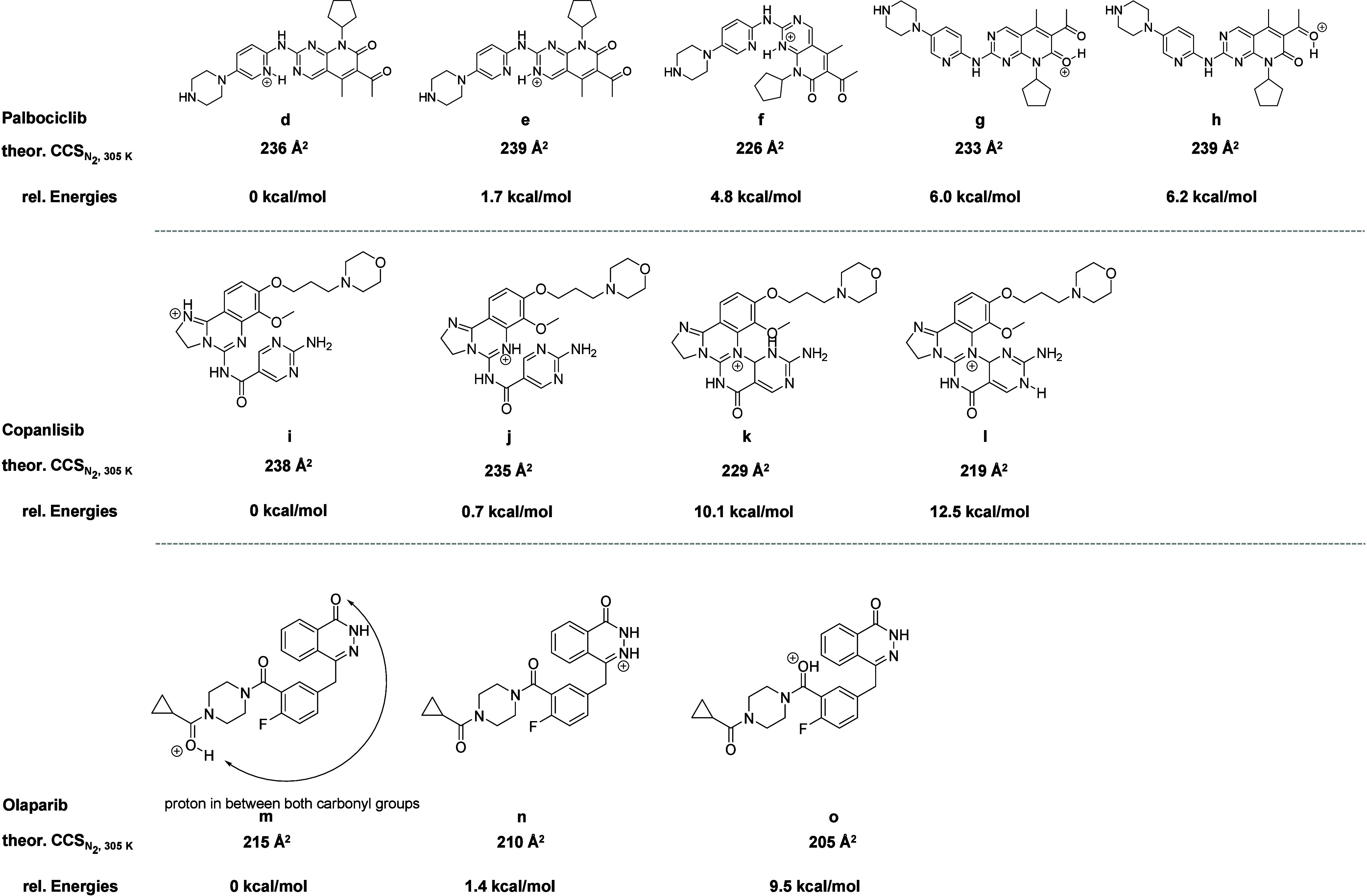
Chemical Structures of the Energy- and Geometry-Optimized Protomers of the Drugs with Their Calculated CCS Values

Next, we matched experimental results with theoretical CCS values, which were calculated using the trajectory method using the high-performance collision cross section (HPCCS) software.^39^ For palbociclib, we found that the theoretical CCS for structures f and gagree well with the experimental CCS values for peaks 1 and 2 of the TIMS-MS analysis (Scheme 2). For copanlisib, the calculated CCS values for structures k and l were 229 and 219 Å^2^, respectively, which matched the experimental CCS values. This further suggests that there are two stable protomers generated for copanlisib during ionization, exhibiting chemical structures similar to structures k and l, respectively. Since we were not able to separate any protomers for olaparib, we speculate that the possible conformation of the olaparib ion was structure o, as the theoretical CCS closely matched the experimental value. A more detailed computational study to shed further light on these assignments is shown in the following section.

**Figure 2:**
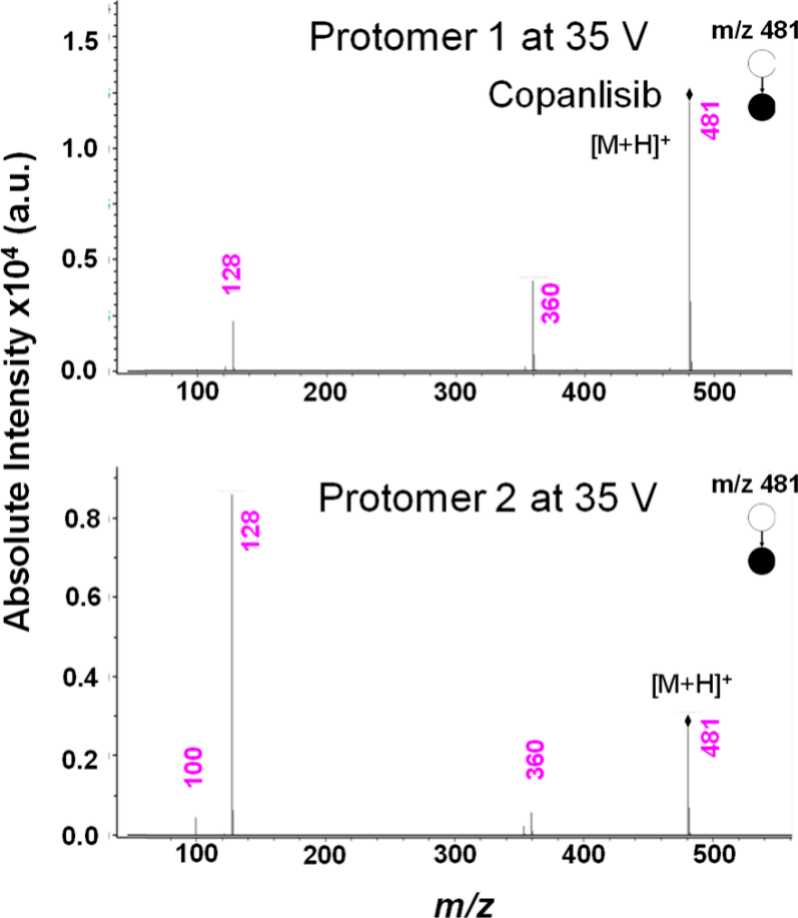
Comparative MS/MS spectra of protomers **1** (top) and **2** (bottom) of copanlisib at 35 V collision offset voltage.

Based on the above observation, we performed CID of TIMS-separated isomers for copanlisib because it provided sufficient signal intensities and good separation in TIMS. Figure 2 presents a comparative MS/MS spectrum. Both protomers yield similar fragmentation patterns, resulting in two fragment ions at *m*/*z* 128 and 360, respectively. However, we noticed that the major fragment ion for protomer 1 was *m*/*z* 360, whereas that for protomer 2 was *m*/*z* 128. We also noticed an additional product ion at *m*/*z* 100 for protomer 2 (we propose a fragmentation pathway in the Supporting Information, Figure S5).

**Figure 3:**
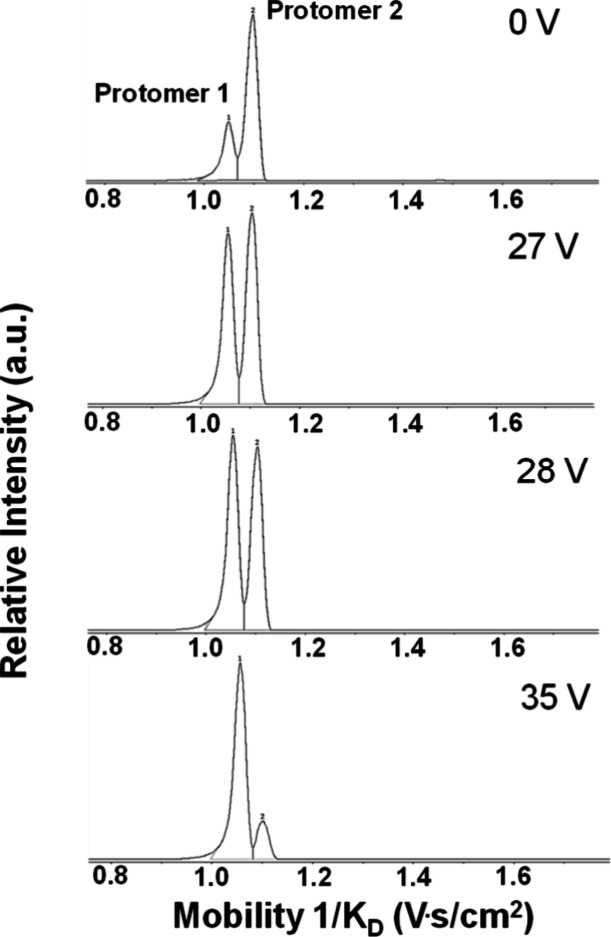
TIMS arrival time distribution of both protomers of copanlisib as a function of the collision offset voltage between 0 and 35 V.

Careful observation of the fragmentation behavior also showed that protomer 1 is more stable than protomer 2 upon CID. In Figure 2, it is noticeable that at a constant collision offset voltage of 35 V, the relative intensity of the precursor ion signal for protomer 1 is higher than that for the dissociated species, in contrast to the CID MS of protomer 2. We then gradually increased the collision energy from 0 to 35 V to observe a systematic change in the TIMS spectra of copanlisib. Figure 3 shows that upon an increase in the collision energy, the relative intensity of the protomer 2 peak decreases in comparison to protomer 1.

### Computational Study

In order to elucidate the structures of the proposed protomers described in the previous section, we calculated both solution- and gas-phase basicities for the various functional groups of the molecules (Tables 1–3). For palbociclib, preliminary gas-phase calculations suggested that the basicity for protonation at O-1 (amide) is 10.6 kcal·mol^–1^ higher than that for protonation at the most basic atom of the piperazinyl group (N-4/N-5). Although protonation at oxygen atoms usually results in lower gas basicity (GB) compared to protonation at nitrogen atoms, the gas-phase basicity (GB) of the —C=O group is relatively high due to the presence of intramolecular hydrogen bonding (IHB) in the protonated form. However, if we additionally consider the relatively free rotation around the C–N bond (Supporting Information, Figure S6), calculations predict that the 2-amino-pyridine group (N-3) exhibits even higher GB, with IHB contributing to the increased GB (H-bonding between N-2/N-3, see Supporting Information, Figure S6). The same holds true for protonation at the N-2 position (pyrimidine moiety). Nevertheless, the GB at O-1 remains relatively high and is very close to the GB for protonation at the N-2 and N-3 positions, primarily due to contribution of IHB. There is, however, repulsion of electron pairs of the two O atoms in the neutral (nonprotonated) form. As a result, the —CH_3_—C=O group does not lie in a plane with the remainder of the palbociclib molecule but instead is substantially twisted. This twisting disrupts the conjugation of the π-bonds, which destabilizes the neutral form. Upon protonation, however, this repulsion disappears and the —CH_3_—C=O group falls in plane with the rest of the molecule, contributing further to the high gas-phase basicity.

**Table 1:**
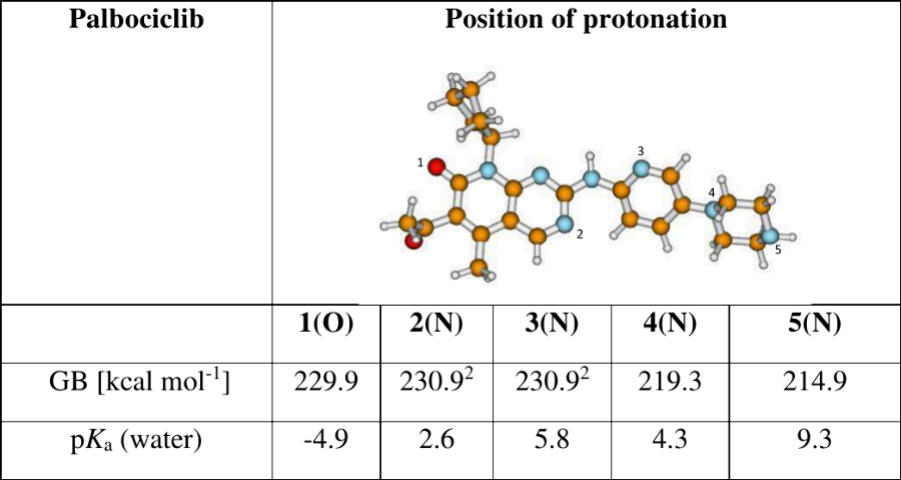
Calculated Solution- (p*K*_a_) and Gas-Phase Basicity (GB) for Palbociclib^1^

1 Model: SMD(M062*X*/6-311++G(3df,2p)//M062X//M062*X*/6-311+G(d,p). The reference base for solution-phase p*K*_a_ was piperazine, p*K*_a_ = 9.72.

2 Conformer, where an intramolecular hydrogen bond is formed in the protonated form between N(2)–H···N(3) and N(3)–H···N(2) upon rotation around the N(H)–C bond.

**Table 2:**
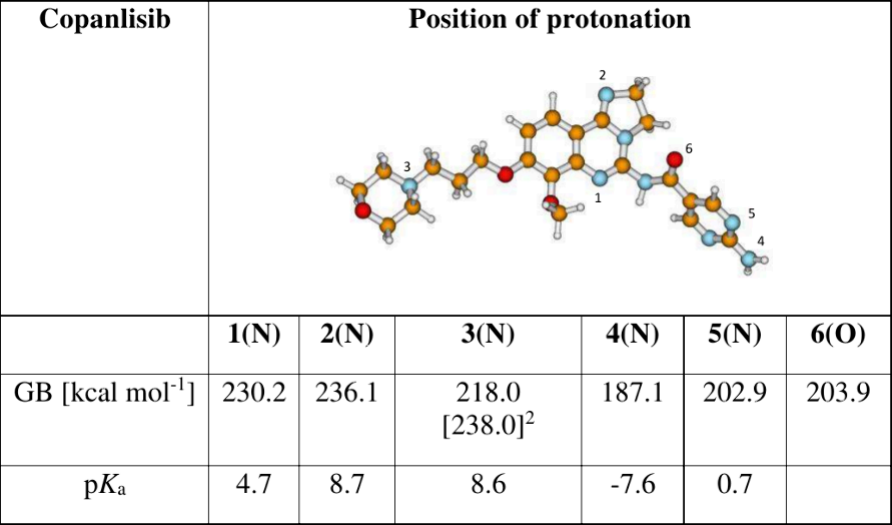
Calculated Solution- (p*K*_a_) and Gas-Phase Basicity (GB) for Copansilib^1^

1 Model: SMD(M062*X*/6-311++G(3df,2p)//M062X//M062*X*/6-311+G(d,p). The reference base for solution-phase p*K*_a_ was piperazine, p*K*_a_ = 9.72.

2 Conformer, where an intramolecular hydrogen bond is formed in the protonated form between N(3)–H and the methoxy group (see Figure S7).

**Table 3:**
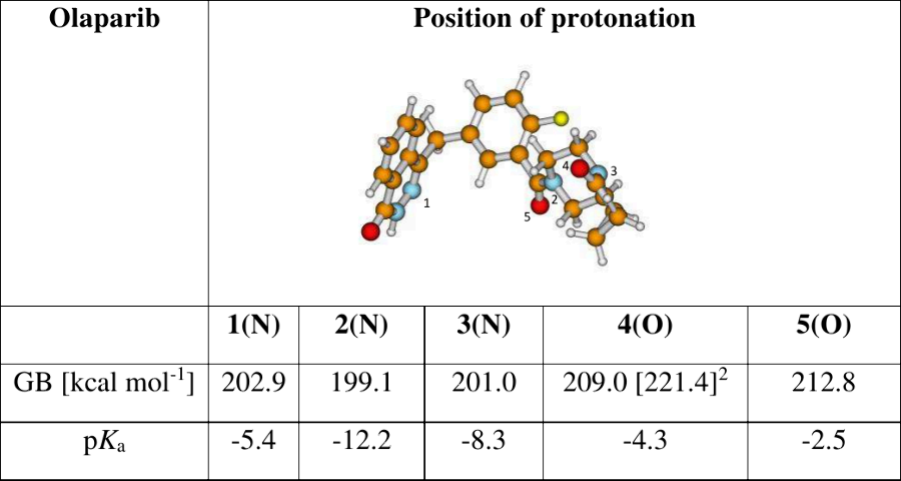
Calculated Solution- (p*K*_a_) and Gas-Phase Basicity (GB) for Olapirib^1^

1 Model: SMD(M062*X*/6-311++G(3df,2p)//M062X//M062*X*/6-311+G(d,p). The reference base for solution-phase p*K*_a_ was piperazine, p*K*_a_ = 9.72.

2 Conformer, where an intramolecular hydrogen bond is formed in the protonated form between 4(O)–H and the O atom of the 3(2*H*)-pyridazinone group.

By comparing the gas-phase basicities of the three drugs, it is evident that both palbociclib and copanlisib exhibit protomer structures, the energies of which are very similar (NB: the differences in the gas phase basicity values represent the differences in stability between two protomers). Both drugs have protomer structures with an energy difference of less than 2 kcal·mol^–1^ (Tables 1 and 2). (Note: the most stable protomer for copanlisib in our calculations was the structure with an intramolecular hydrogen bond; see Supporting Information, Figure S7. This structure was not obtained from the CCS calculations using the commercial instrument software (Scheme 2); this species likely has a different CCS value).

As a result, the two relatively basic sites for these two drugs provide two distinct charge locations for protomer formation in the gas phase, supporting the results from the ion mobility spectrometry investigations described in the previous section. Only one major protomer was observed for olaparib, however, which agrees well with the computational data, illustrating a larger energy difference between the most stable protomer and the next species at 8.4 kcal·mol^–1^ (GB = 221.4–212.8 kcal·mol^–1^, Table 3).

However, as electrospray ionization was employed, solution-phase behavior must also be considered, as pre-formed ions are often transferred into the gas-phase in ESI and mass spectra may therefore (partially) represent solution-phase behavior.^40^

In aqueous solution, for palbociclib, the piperazine moiety (N-5, calculated p*K*_a_ = 9.3) is the most basic group, which was expected, as the p*K*_a_ for the piperazine molecule in water is 9.73.^37^ The charged site N-3 can be approximated by 2-amino pyridine, the experimental p*K*_a_ of which is 6.86.^41^ In aqueous solution, IHBs are usually disrupted by interactions of water molecules with the basic sites; thus, the p*K*_a_ was not expected to increase via IHB. The calculated p*K*_a_ values for N-2 and N-3 were 2.6 and 5.8, respectively (Table 1). A similar p*K*_a_ value (8.7) was obtained for copanlisib (Table 2). As a result, both palbociclib and copanlisib will be protonated in aqueous solution under the chosen experimental conditions. Please note that copanlisib exhibits two sites in the molecule with virtually equal p*K*_a_ values, suggesting that both protonated forms may exist in solution. Importantly, olaparib is not basic (Table 3; all calculated p*K*_a_ values are well below 0); that is, it cannot be protonated in an aqueous solution under the chosen experimental conditions.

Considering both the gas-phase and solution-phase basicities of the three molecules, we suggest the following two mechanistic scenarios for the formation of two protomers for palbociclib and copanlisib, but only one protomer for olaparib:

(1) If complete “reorganization” of protonated variants in the gas phase is possible, only the most stable protonated forms are present in the gas phase, and these should have very similar basicities (i.e., similar stabilities). (Reorganization refers to proton being able to move easily and quickly from one position to another in the gas phase during the experiment).
(2) If the protonated site in solution is different from the protonated site in the gas phase and reorganization of the protonated form in the gas phase is not completely possible, then the most stable protonated form from the solution will also (partially) be present in the gas phase.

If we apply scenario 2, we would conclude that palbociclib and copanlisib (both with basic groups of high p*K*_a_ values) both generate stable protomers in solution and transfer them into the gas phase. As a result, these protomers will also be present in the gas phase together with protomers, where the proton resides at the most basic site in the gas phase. Olaparib is not basic and cannot be protonated in solution in our experiments (Table 3).

However, if proton reorganization in the gas phase is readily possible (scenario 1), the proton can move from the most stable position in solution to the most stable positions in the gas phase upon ionization. As shown above, palbociclib and copanlisib exhibit two protomers with very similar stabilities, whereas olaparib exhibits only one high-basicity site in the gas phase, explaining the observed single protomer. In addition, it cannot transfer a preformed ion from solution to the gas phase, as explained above.

Please note that both palbociclib and copanlisib are basic in solution and that the most basic site in solution is different from that in the gas phase. If reorganization is not complete, the most stable protomer from solution may also be present to some extent in the gas phase. If reorganization is complete, we still have two protomers with similar stabilities.

In conclusion, both scenarios 1 and 2 readily connect the observed experimental and computational data, and our data cannot distinguish between the two mechanisms.

We also calculated basicities for doubly protonated palbociclib, which was seen in our analyses (see Supporting Information, Figure S1). It appears that the most stable doubly protonated species in aqueous solution has protons located at N-5 and N-2 (Table 1). However, the calculated p*K*_a_ for the second protonation step has a value of −6.1, thus ruling out this proton addition in an aqueous solution under regular experimental conditions. In the gas phase, however, the situation is different: the most stable doubly protonated species has the protons located at N-5 and O-1. The gas-phase basicity for the second protonation step is 173.7 kcal·mol^–1^, which compares well with the experimental gas basicity of methanol (173.2 kcal·mol^–1^). It is therefore conceivable that the observed [M + 2H]^2+^ ion of palbociclib in our mass spectra is generated during ESI after protonation at N-5 in solution and the addition of the second proton in the gas phase to give the doubly protonated [M + 2H]^2+^ species.

## Conclusion

We demonstrate that the ionization of molecules such as palbociclib, copanlisib, and olaparib results in the formation of isomers due to protonation at different positions within the molecules. These protomers show discrete properties such as dissimilar fragmentation patterns during MS analysis. Using different analytical techniques, such as mass spectrometry and ion mobility spectrometry, we detected two protomers for palbociclib and copanlisib. However, olaparib did not exhibit isomeric separation, suggesting the dominance of one protomer over others in the gas phase. We believe that our work will have implications for the quantification of these drugs using LC-MS/MS, where the quantification relies on the highly reproducible intensity distribution of the fragmented species of isolated mass-selected precursor ions.

## Supporting Information Available

Full scan mass spectra, MS/MS spectra, DT IMS spectra, fragmentation pathway, gas-phase structures of the drugs, and gas-phase geometries of neutral and protonated species (PDF)
