# Supplementary material for: Characterization of Anticancer Drug Protomers Using Electrospray Ionization and Ion Mobility Spectrometry–Mass Spectrometry
Source: J Am Soc Mass Spectrom. 2024 Sep 27;35(12):2869–76. doi: 10.1021/jasms.4c00233 (PMC11622236; doi:10.1021/jasms.4c00233)
Supplement: Supplementary file 1 — js4c00233_si_001.pdf [file js4c00233_si_001.pdf]

Supplementary Information

Characterization of Anticancer Drug Protomers Using Electrospray Ionization and Ion Mobility Spectrometry-Mass Spectrometry

Pallab Basuri[1] Marc Safferthal,[2] Borislav Kovacevic,[3] Pascal Schorr,[1] Jerome Riedel,[2] Kevin Pagel,[2] Dietrich A. Volmer[1]*

[1]*Institute of Chemistry, Humboldt-Universität zu Berlin, 12489 Berlin, Germany.*

[2]*Institute of Chemistry and Biochemistry, Freie Universität Berlin, 14195 Berlin, Germany.*

[3]*Division of Physical Chemistry, Ruđer Bošković Institute, 10000 Zagreb, Croatia.*

*Corresponding author:

Prof. Dr. Dietrich Volmer

Humboldt University Berlin

Department of Chemistry

12489 Berlin, Germany

Tel +49 30 2093 7588

Email: Dietrich.Volmer@hu-berlin.de

Table of contents

Description	Page
**Figure S1.** MS of Palbociclib and Copanlisib using Sciex QTRAP 6500+ MS ………………….	3
**Figure S2.** MSMS of doubly change species of Palbociclib and Copanlisib ……………………	4
**Figure S3.** MS of Olaparib using Sciex QTRAP 6500+ MS ……………………………………….	5
**Figure S4.** DT IMS of the drugs using synapt g2-si mass spectrometer ………………………..	6
**Figure S5.** Fragmentation pathway of protomer 2 of Copanlisib to m/z 100 …………………....	7
**Figure S6.** Gas phase structures of protonated palbociclib ……………………………………….	8
**Figure S7.** Copanlisib protonated at N(3) with intramolecular H-bond. ………………………….	9
**Table S1.** Gas phase geometries of neutral and protonated species …………………………..	10

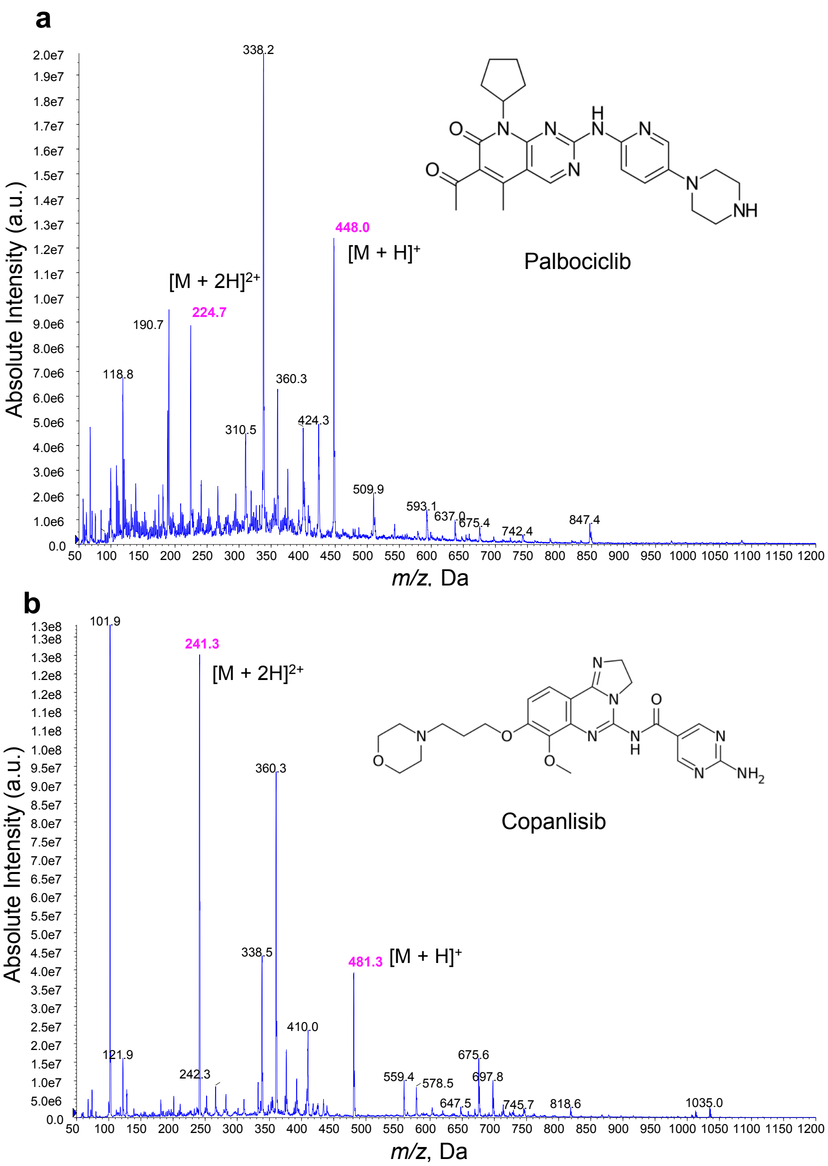

Figure S1. ESI MS characterization of the drugs a) palbociclib and b) copanlisib using QTRAP 6500+ triple quadrupole mass spectrometer. All the other peaks correspond to the background signal.

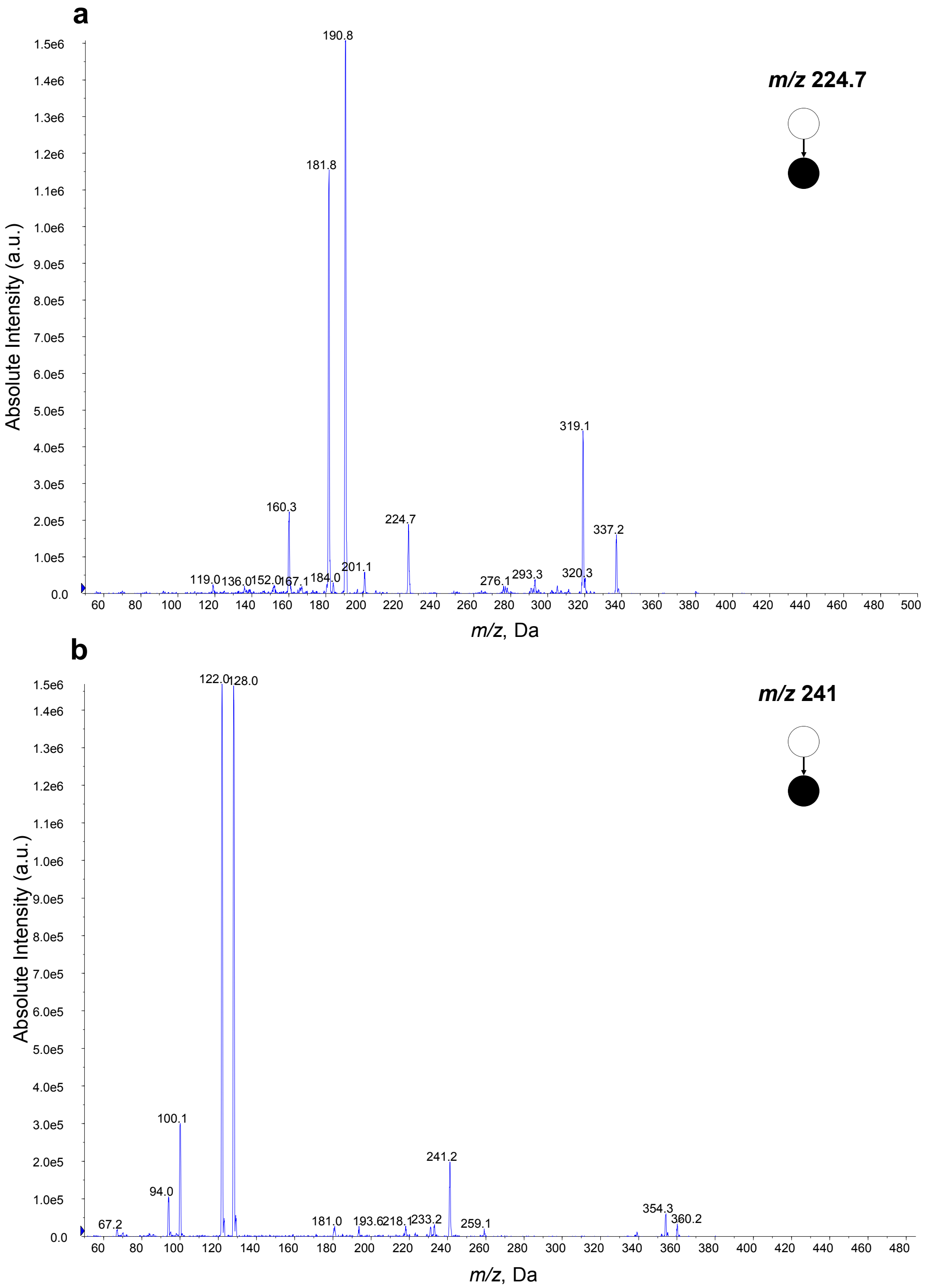

Figure S2. MSMS of the isolated species at a) m/z 224.7 and b) m/z 241, corresponding to the doubly charged ion of palbociclib and copanlisib, respectively.

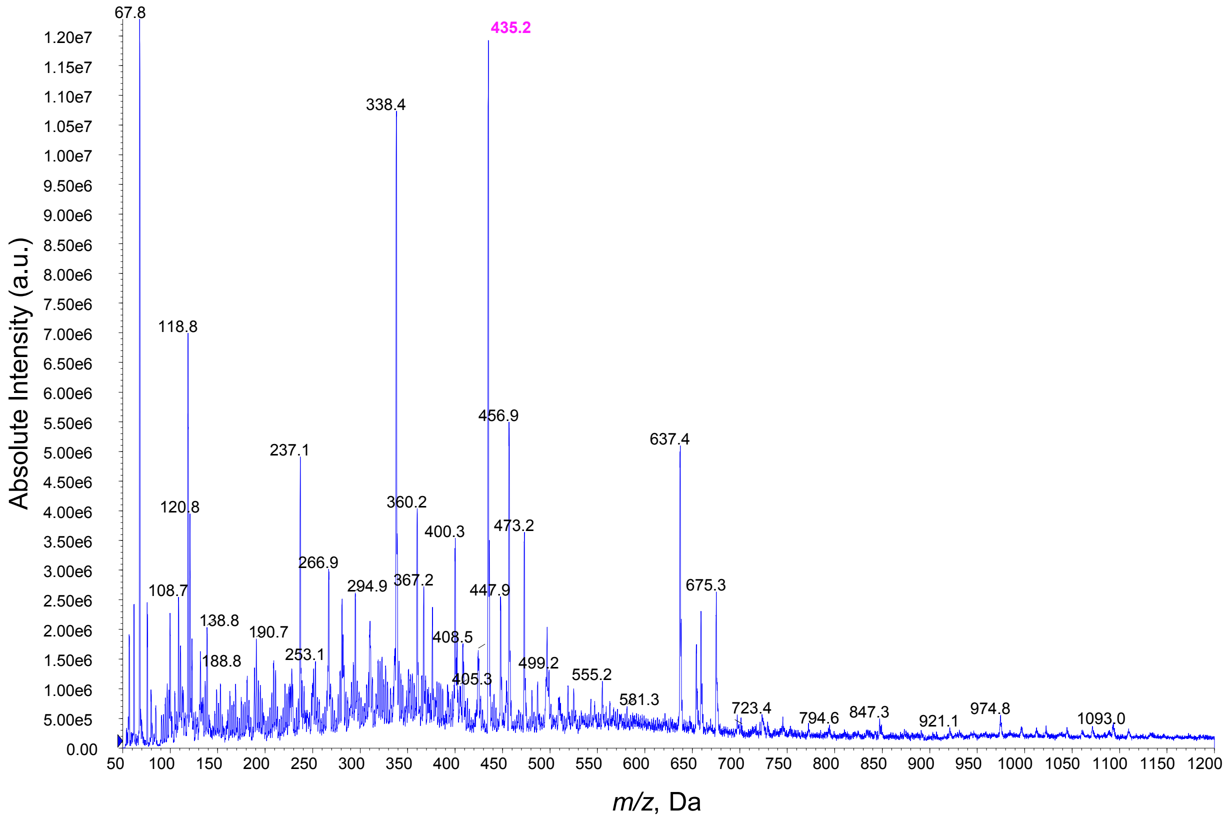

Figure S3. ESI MS characterization of olaparib using QTRAP 6500+ triple quadrupole mass spectrometer where no doubly charged species was observed. All the other peaks correspond to the background signal.

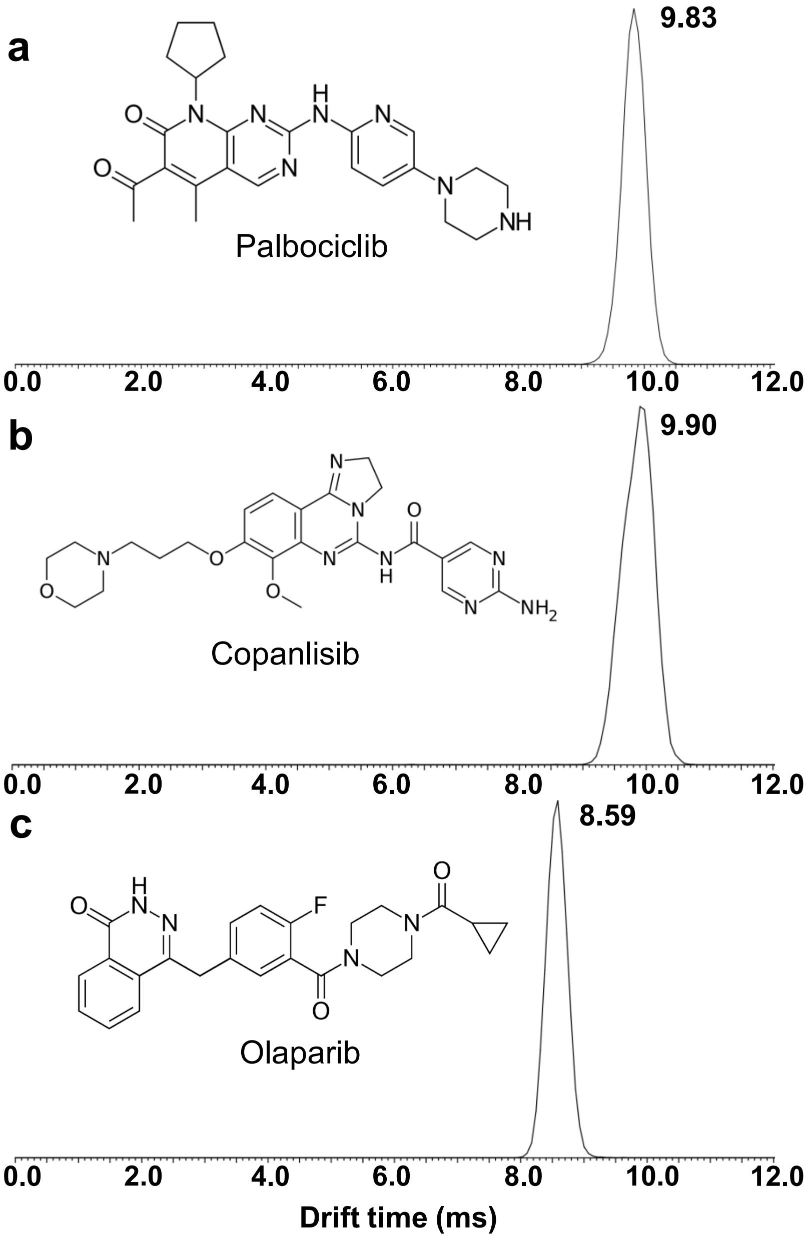

Figure S4. DT IMS of a) palbociclib, b) copanlisib and c) olaparib using Synapt Drift Tube IMS using N_2 nebulization.

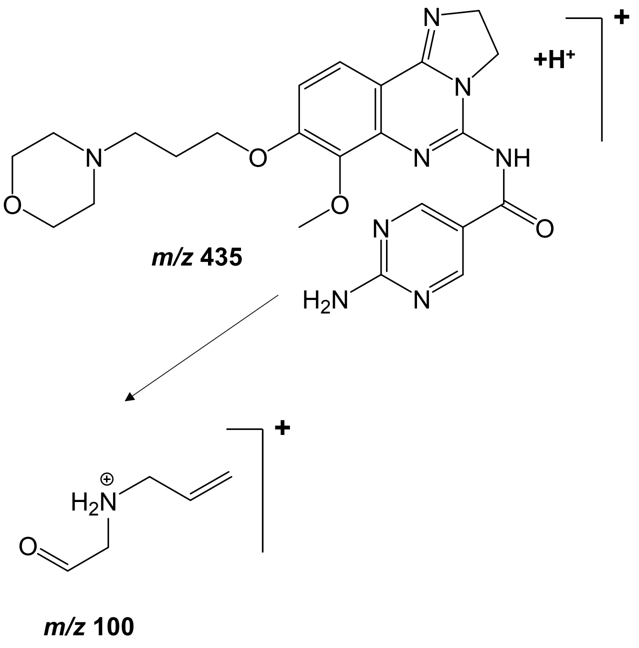

Figure S5. The Fragmentation pathway of one of the protomer leading to product peak at m/z 100.

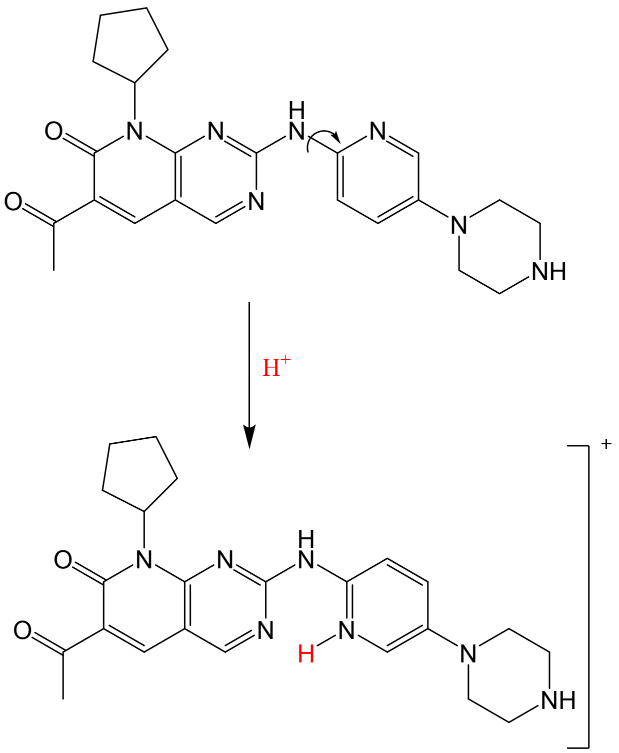

Figure S6. Free rotation around the C–N bond of palbociclib in the gas phase after protonation.

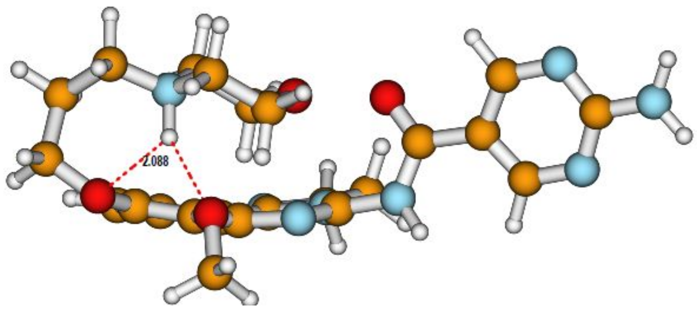

Figure S7. Copanlisib protonated at N(3) with intramolecular H-bond.

Table S1. Gas phase geometries of neutral and protonated species optimized utilizing M06-2X/6-311+G(d,p) level of theory

Palbociclib-neut

Atomic Number	Coordinates (Angstroms) X	Y	Z
6	3.488933	2.095563	0.166210
6	3.878414	2.766624	-1.166576
6	4.559200	4.065453	-0.722906
6	5.382081	3.625779	0.495086
7	3.471510	0.617422	0.089108
6	4.692595	-0.049209	-0.071292
6	4.673322	-1.517988	-0.111174
6	3.507195	-2.219488	-0.078579
6	2.273219	-1.477240	0.001598
6	2.276032	-0.061881	0.080116
6	1.003452	-2.056728	-0.022461
7	-0.130132	-1.379227	0.035013
6	-0.000243	-0.044703	0.121513
7	1.150435	0.637086	0.141552
8	5.722836	0.592689	-0.177597
6	3.427936	-3.721954	-0.102246
6	6.012135	-2.199310	-0.212779
8	6.151628	-3.157124	-0.938414
7	-1.113914	0.732295	0.192231
6	-2.481691	0.420563	0.179610
7	-3.244055	1.516180	0.227099
6	-4.559191	1.377470	0.231908
6	-5.221739	0.143245	0.170084
6	-4.406079	-0.987595	0.125879
6	-3.021792	-0.862711	0.139248
7	-6.630685	0.107035	0.195426
6	-7.236049	-1.201847	0.408084
6	-8.723168	-1.040231	0.690352
7	-9.350106	-0.356379	-0.433895
6	-8.774541	0.971329	-0.611884
6	-7.288769	0.827995	-0.901232
6	7.156656	-1.695595	0.633805
1	0.901614	-3.134364	-0.102646
1	2.719240	-4.072483	0.649875
1	3.078381	-4.060234	-1.081628
1	4.393175	-4.184382	0.072775
1	-0.934836	1.726546	0.241497
1	-5.135097	2.295118	0.305041
1	-4.837275	-1.979443	0.079105
1	-2.379366	-1.727231	0.108187
1	-7.110336	-1.852216	-0.473358
1	-6.752007	-1.675086	1.265116
1	-9.176612	-2.026507	0.807103
1	-8.838297	-0.486758	1.635887
1	-9.260818	1.462022	-1.457306

1	-8.896782	1.604909	0.281247
1	-7.155390	0.281192	-1.847983
1	-6.837009	1.813561	-1.008105
1	4.595663	2.142329	-1.701087
1	3.007084	2.921970	-1.805256
1	3.806045	4.801656	-0.421016
1	5.164183	4.513378	-1.512563
1	5.713900	4.463406	1.110593
1	6.259811	3.071348	0.158400
1	3.840094	3.230570	1.975262
1	4.977607	1.898352	1.790032
1	2.467770	2.364905	0.412325
1	-10.352185	-0.295567	-0.299671
1	7.811547	-2.538143	0.853459
1	7.707762	-0.945051	0.065472
1	6.809612	-1.217886	1.549995

Palbociclib-prot_N2

Atomic Number	Coordinates (Angstroms) X	Y	Z
6	4.578765	2.506832	1.372128
6	3.691781	2.065268	0.174540
6	4.326355	2.663285	-1.097047
6	5.118476	3.859374	-0.557085
6	5.722498	3.319373	0.745045
7	3.476103	0.593601	0.080049
6	4.629517	-0.237310	0.034223
6	4.434907	-1.696631	-0.028153
6	3.205509	-2.256026	-0.066430
6	2.061023	-1.357938	-0.046838
6	2.232301	0.072056	0.019869
6	0.769174	-1.801778	-0.067253
7	-0.266896	-0.936292	-0.044601
6	-0.030649	0.399553	-0.005569
7	1.188607	0.903262	0.026899
8	5.722845	0.270174	0.066399
6	2.939030	-3.732691	-0.140975
6	5.698033	-2.539795	-0.016837
6	6.824750	-2.174925	-0.945274
7	-1.076353	1.248231	0.000858
6	-2.459475	0.951056	-0.020154
7	-2.825289	-0.325591	-0.042595
6	-4.121942	-0.627807	-0.068664
6	-5.149095	0.331514	-0.065933
6	-4.727991	1.674690	-0.033913
6	-3.382065	1.989789	-0.018696
7	-6.479488	-0.045928	-0.134151
6	-7.477197	1.017210	-0.259057
6	-8.818537	0.435552	-0.683520
7	-9.224374	-0.576696	0.277621
6	-8.274044	-1.677045	0.279713

6	-6.922341	-1.149477	0.733910
8	5.747051	-3.493691	0.717216
1	0.507586	-2.850714	-0.096327
1	7.377185	-3.081712	-1.186994
1	6.465348	-1.683387	-1.849948
1	7.487993	-1.475628	-0.432907
1	2.507153	-4.082680	0.801027
1	2.234448	-3.951655	-0.946670
1	3.847647	-4.300617	-0.307666
1	-0.807859	2.222857	0.030282
1	-4.352405	-1.683998	-0.116270
1	-5.449935	2.478733	-0.007013
1	-3.058093	3.023281	0.008475
1	-7.598293	1.553840	0.694124
1	-7.144431	1.720110	-1.024668
1	-9.557973	1.237491	-0.701990
1	-8.716875	0.031601	-1.703447
1	-10.162783	-0.905251	0.085467
1	-8.606303	-2.443225	0.981911
1	-8.159304	-2.138232	-0.714474
1	-7.005705	-0.789010	1.768774
1	-6.194177	-1.957434	0.706416
1	5.012476	1.947843	-1.551832
1	3.569548	2.934001	-1.835229
1	4.441886	4.692966	-0.341575
1	5.867614	4.215030	-1.264576
1	6.081326	4.108151	1.406285
1	6.558402	2.657401	0.513647
1	3.977488	3.138946	2.029088
1	4.936679	1.666279	1.964754
1	2.697148	2.476234	0.301669
1	-1.273808	-1.201986	-0.055994

Palbociclib-prot_N3

Atomic Number	Coordinates (Angstroms) X	Y	Z
6	6.881617	1.339961	-0.287136
7	6.509385	-0.069822	-0.154654
6	7.404410	-0.819997	0.747150
6	8.852862	-0.642941	0.321271
7	9.159954	0.778036	0.312660
6	8.347867	1.463593	-0.678138
6	5.160329	-0.357346	-0.085417
6	4.178605	0.616261	-0.045201
7	2.859994	0.281645	-0.056214
6	2.408860	-0.974841	-0.071315
6	3.361040	-2.010134	-0.095997
6	4.693400	-1.702613	-0.109595
7	1.066055	-1.229315	-0.069030
6	0.007980	-0.320187	-0.038046
7	0.305221	0.978797	-0.021334

6	-0.751946	1.799487	-0.001237
6	-2.063960	1.359173	0.005506
6	-2.240513	-0.054891	0.006829
7	-1.185343	-0.877597	-0.026076
7	-3.487744	-0.591508	0.044775
6	-4.636524	0.231399	0.071583
6	-4.439885	1.690243	0.063194
6	-3.208323	2.252290	0.032955
6	-3.693690	-2.063980	0.058421
6	-4.533242	-2.581665	1.259646
6	-5.698480	-3.360634	0.629793
6	-5.143569	-3.819949	-0.724210
6	-4.375955	-2.589848	-1.220712
8	-5.733913	-0.276320	0.116354
6	-5.701582	2.529267	0.119640
8	-5.749402	3.459065	0.885512
6	-2.950247	3.732959	0.010043
6	-6.839518	2.194326	-0.808068
1	-0.526287	2.858962	0.016807
1	-7.387789	3.110491	-1.022986
1	-6.491792	1.721562	-1.727056
1	-7.502063	1.487180	-0.305973
1	-2.511701	4.050523	0.960194
1	-2.250063	3.982614	-0.790014
1	-3.862448	4.302385	-0.129833
1	0.776126	-2.197912	-0.078419
1	4.379564	1.675280	-0.006631
1	5.410374	-2.511051	-0.168795
1	3.026225	-3.038965	-0.125117
1	7.281733	-0.448433	1.773575
1	7.146998	-1.877598	0.726377
1	9.494499	-1.149901	1.043262
1	8.994443	-1.113358	-0.664751
1	10.146579	0.938693	0.150981
1	8.615542	2.520797	-0.701800
1	8.475504	1.048984	-1.690566
1	6.710382	1.877702	0.658088
1	6.268089	1.787514	-1.072356
1	-5.081204	-1.851601	-1.604148
1	-3.647855	-2.812122	-2.002846
1	-4.457055	-4.661795	-0.583786
1	-5.918565	-4.137332	-1.422244
1	-6.032236	-4.188433	1.255875
1	-6.541967	-2.687427	0.469012
1	-3.907432	-3.248067	1.857649
1	-4.871592	-1.776681	1.910201
1	-2.693941	-2.478084	0.121376
1	2.114580	1.002865	-0.037625

Palbociclib-prot_N4

Atomic Number	Coordinates (Angstroms) X	Y	Z

6	7.192118	-0.338847	1.295825
7	6.610850	0.199371	0.006464
6	7.201206	-0.540424	-1.166985
6	8.699273	-0.178612	-1.264259
6	8.711592	-0.512613	1.110516
6	5.147155	0.265991	0.010233
6	4.526929	1.503748	0.012274
7	3.213115	1.642279	0.018030
6	2.445069	0.543799	0.020525
6	2.993782	-0.759804	0.018152
6	4.364887	-0.886495	0.015002
7	1.104538	0.825786	0.024049
6	-0.014613	0.002107	0.014955
7	0.150504	-1.318346	0.022166
6	-0.979783	-2.023138	0.022570
6	-2.250018	-1.460833	0.013714
6	-2.283252	-0.041531	-0.013405
7	-1.156750	0.673140	-0.002740
7	-3.478405	0.617921	-0.054401
6	-4.698899	-0.082432	-0.056824
6	-4.648579	-1.552875	-0.024548
6	-3.476100	-2.233365	0.008745
6	-3.531229	2.100528	-0.096154
6	-4.324849	2.681114	-1.299681
6	-5.395660	3.589611	-0.675966
6	-4.780867	4.014655	0.663491
6	-4.140988	2.720533	1.177283
8	-5.744621	0.531251	-0.102263
6	-5.983859	-2.263767	-0.053623
8	-6.130796	-3.212770	-0.784656
6	-3.367308	-3.732253	0.059850
6	-7.083386	-1.788292	0.860816
1	-0.861375	-3.101025	0.023180
1	-7.717598	-2.639585	1.104642
1	-6.689947	-1.320460	1.763608
1	-7.673338	-1.036162	0.334523
1	-2.980157	-4.110160	-0.890489
1	-2.677824	-4.033218	0.851209
1	-4.328504	-4.205386	0.228012
1	0.888627	1.815292	0.019117
1	5.109887	2.424047	0.008707
1	4.809042	-1.876422	0.012799
1	2.341133	-1.618009	0.019382
1	7.039877	-1.601183	-0.973265
1	6.639102	-0.256108	-2.054448
1	6.678653	-1.274829	1.513487
1	6.942567	0.392323	2.064317
1	-4.915326	2.067177	1.580779
1	-3.384643	2.880006	1.947913
1	-4.010886	4.776270	0.498900
1	-5.510729	4.426253	1.361378
1	-5.649440	4.435610	-1.315583

1	-6.301809	3.009821	-0.493031
1	-3.639554	3.266898	-1.916837
1	-4.753349	1.903085	-1.929471
1	-2.494496	2.407136	-0.175858
1	6.976854	1.156498	-0.062994
7	9.106490	0.374665	0.024453
1	10.092681	0.600400	0.042369
1	8.857622	0.574110	-2.037117
1	9.254782	-1.082400	-1.543794
1	9.220469	-0.228505	2.030055
1	8.952925	-1.563882	0.902703

Palbociclib-prot_N5

Atomic Number	Coordinates (Angstroms) X	Y	Z
6	-4.716165	0.092139	0.057744
7	-3.499123	-0.609982	0.054682
6	-2.298718	0.045900	0.012477
6	-2.263707	1.464220	-0.015438
6	-3.486742	2.238485	-0.011452
6	-4.662774	1.561933	0.022615
6	-0.989472	2.021438	-0.023991
7	0.136919	1.315732	-0.023525
6	-0.027373	-0.008743	-0.017145
7	-1.177833	-0.673100	0.001534
1	-0.868040	3.099321	-0.023985
7	1.082680	-0.828735	-0.028186
6	-3.374242	3.737267	-0.066615
6	-5.994140	2.276570	0.046568
8	-5.764608	-0.519557	0.106269
6	-3.554247	-2.091356	0.098461
8	-6.139003	3.237724	0.763221
6	-7.099534	1.792522	-0.857324
1	-7.730821	2.643389	-1.110345
1	-6.711630	1.310202	-1.754774
1	-7.690344	1.050002	-0.318672
1	-2.994100	4.117004	0.885812
1	-2.676656	4.033562	-0.852463
1	-4.332714	4.212355	-0.244448
6	2.435813	-0.551759	-0.025003
1	0.869239	-1.818129	-0.022663
7	3.188136	-1.658111	-0.023222
6	4.506795	-1.532249	-0.017336
6	5.158105	-0.305554	-0.012765
6	4.369827	0.846040	-0.016053
6	2.992463	0.741480	-0.022698
1	5.089140	-2.449386	-0.015611
7	6.597361	-0.276738	-0.003845
1	4.824766	1.832528	-0.013117
1	2.350435	1.607237	-0.025054
6	7.165960	0.304383	1.201803

6	8.652457	-0.003380	1.262797
1	7.010473	1.397179	1.260324
1	6.688209	-0.140600	2.075353
7	9.311707	0.538072	0.015860
1	9.140354	0.457699	2.121174
1	8.826884	-1.079388	1.261677
6	8.668690	0.002877	-1.242166
1	10.310959	0.319786	0.021798
6	7.181468	0.310364	-1.199029
1	9.167716	0.468131	-2.091840
1	8.843077	-1.073139	-1.244094
1	7.026829	1.403463	-1.254211
1	6.715171	-0.130349	-2.080894
6	-4.167286	-2.712747	-1.172748
6	-4.808252	-4.005208	-0.656315
6	-5.419720	-3.577377	0.683879
6	-4.346079	-2.669902	1.304091
1	-4.941281	-2.058590	-1.575661
1	-3.412205	-2.874492	-1.944150
1	-4.038943	-4.767623	-0.491909
1	-5.540204	-4.416968	-1.352118
1	-5.674270	-4.422203	1.324986
1	-6.324722	-2.995516	0.501552
1	-3.660406	-3.256034	1.920613
1	-4.772380	-1.890264	1.933351
1	-2.517683	-2.399103	0.176274
1	9.234319	1.560570	0.017947

Palbociclib-prot_O

Atomic Number	Coordinates (Angstroms) X	Y	Z
6	-4.620410	0.043544	0.029830
7	-3.459782	-0.633057	0.109705
6	-2.254408	0.061726	0.103001
6	-2.283077	1.481557	0.137774
6	-3.494507	2.201664	0.098447
6	-4.682346	1.480433	-0.043417
6	-0.990831	2.080999	0.168366
7	0.123600	1.413125	0.150828
6	0.016617	0.053594	0.110061
7	-1.143788	-0.633474	0.090348
1	-0.893845	3.162036	0.185736
7	1.122498	-0.684694	0.084859
6	-3.445906	3.698091	0.255456
6	-6.032629	2.047137	-0.303315
8	-5.692110	-0.674439	0.027369
6	-3.459049	-2.130017	0.176130
8	-7.017528	1.316619	-0.201055
6	-6.265836	3.453199	-0.779944
1	-7.194649	3.443777	-1.347852
1	-6.409481	4.118282	0.075593

1	-5.453850	3.834689	-1.394439
1	-2.644371	3.977857	0.936066
1	-3.257305	4.187755	-0.704282
1	-4.367614	4.091387	0.669083
6	2.503653	-0.361159	0.083051
1	0.972493	-1.687804	0.045304
7	3.247053	-1.462704	0.004437
6	4.561552	-1.347410	0.000416
6	5.243838	-0.112972	0.067914
6	4.431974	1.025811	0.150010
6	3.047632	0.913091	0.165590
1	5.113414	-2.279161	-0.034358
7	6.631327	-0.071763	0.097197
1	4.868708	2.014047	0.189858
1	2.421631	1.788245	0.225785
6	7.260774	1.227777	0.318282
6	8.728846	1.046768	0.678977
1	7.181987	1.860816	-0.579242
1	6.754226	1.724051	1.147869
7	9.393324	0.302202	-0.379461
1	9.192156	2.029960	0.776396
1	8.787110	0.535327	1.653079
6	8.814738	-1.028034	-0.498850
1	10.390619	0.249082	-0.211395
6	7.352402	-0.889356	-0.890134
1	9.339074	-1.582115	-1.279164
1	8.877039	-1.600484	0.440522
1	7.288369	-0.411960	-1.878583
1	6.898883	-1.876099	-0.954019
6	-3.945369	-2.793476	-1.127025
6	-4.541124	-4.115796	-0.629925
6	-5.270385	-3.710722	0.657035
6	-4.301047	-2.729831	1.336294
1	-4.727651	-2.196160	-1.598230
1	-3.130243	-2.917298	-1.840890
1	-3.740326	-4.825851	-0.401115
1	-5.194848	-4.579980	-1.368140
1	-5.511581	-4.560509	1.295149
1	-6.202926	-3.201919	0.404822
1	-3.623758	-3.265372	2.003781
1	-4.809612	-1.973453	1.932687
1	-2.414118	-2.371519	0.329795
1	-6.482677	-0.012215	-0.019861

Copanlisib-neut

Atomic Number	Coordinates (Angstroms) X	Y	Z
6	2.870357	0.736774	-0.261588

7	4.049375	-0.011220	-0.455772
7	2.997608	2.077868	-0.112231
6	1.866128	2.916535	-0.037920
6	0.581649	2.236501	-0.039368
6	0.586547	0.836293	-0.198346
7	1.756561	0.092041	-0.302098
6	4.171892	2.934763	-0.323575
7	2.109575	4.165461	-0.008040
6	-0.617130	2.931747	0.074796
6	-0.637000	0.154931	-0.246531
6	5.091326	-0.043415	0.466897
6	6.144868	-1.046153	0.179134
6	7.039364	-1.399376	1.195082
7	8.010714	-2.274916	1.025537
6	8.109241	-2.815694	-0.204006
7	7.330952	-2.534419	-1.263036
6	6.363841	-1.657116	-1.055018
7	9.086924	-3.724758	-0.389841
1	5.753135	-1.414433	-1.921565
1	9.210543	-4.139066	-1.297242
1	9.703793	-3.946848	0.372162
1	6.942128	-0.943036	2.175699
8	5.144429	0.693842	1.425073
1	3.831280	-0.894659	-0.900473
6	3.567787	4.325365	-0.007732
1	3.866453	5.075973	-0.739917
1	3.886215	4.675916	0.978095
1	4.979919	2.659169	0.347904
1	4.500866	2.845668	-1.362721
6	-1.824560	2.254861	0.046846
6	-1.837924	0.861318	-0.107611
8	-0.676476	-1.188034	-0.457889
6	-0.506377	-1.953239	0.728951
1	-0.557752	-2.999797	0.433572
1	0.466934	-1.743570	1.180192
1	-1.307799	-1.733473	1.440996
1	-0.583918	4.008301	0.189174
1	-2.748484	2.806717	0.147551
8	-2.961141	0.108765	-0.131807
6	-4.213131	0.765576	-0.024310
6	-5.292679	-0.296011	-0.100108
1	-4.268815	1.307822	0.928590
1	-4.324098	1.490161	-0.840115
6	-6.668935	0.318087	0.137338
1	-5.269560	-0.771568	-1.083839
1	-5.066375	-1.060805	0.646340
7	-7.762809	-0.568668	-0.232317
1	-6.757477	0.632676	1.194729
1	-6.768065	1.223071	-0.472275
6	-9.043865	0.120753	-0.118465
6	-10.175307	-0.805634	-0.532552
1	-9.220085	0.461297	0.918104
1	-9.032580	0.999825	-0.768888

8	-10.188292	-1.973657	0.262987
1	-11.141676	-0.319815	-0.395301
1	-10.056467	-1.077902	-1.590363
6	-8.959105	-2.661532	0.152677
6	-7.791597	-1.783789	0.576239
1	-9.030867	-3.539710	0.794761
1	-8.808687	-2.987414	-0.885829
1	-7.887818	-1.539944	1.650491
1	-6.863563	-2.340875	0.432843

Copanlisib-prot_N1

Atomic Number	Coordinates (Angstroms) X	Y	Z
6	-7.834397	-1.676730	0.845944
7	-7.790411	-0.553885	-0.089039
6	-9.071925	0.150562	-0.064636
6	-10.203707	-0.812594	-0.383355
8	-10.226517	-1.882591	0.538280
6	-9.005486	-2.588182	0.509901
6	-6.699673	0.361819	0.198957
6	-5.326735	-0.266763	-0.033298
6	-4.246772	0.793873	-0.012788
8	-2.986772	0.126944	-0.157363
6	-1.861679	0.853717	-0.166465
6	-0.665330	0.127484	-0.304299
6	0.535956	0.822238	-0.249011
6	0.592209	2.206107	-0.083989
6	-0.598079	2.918074	0.006427
6	-1.810553	2.248799	-0.032628
7	1.726115	0.077508	-0.332195
6	2.934747	0.634556	-0.229970
7	3.038534	1.940774	-0.087726
6	1.902491	2.834523	-0.059949
7	2.204602	4.056678	-0.071186
6	3.657898	4.197336	-0.143189
6	4.258263	2.771271	-0.051508
7	4.042471	-0.170997	-0.341327
6	5.082514	-0.174454	0.645838
8	4.967710	0.505666	1.631749
8	-0.570792	-1.221138	-0.411075
6	-1.460961	-1.905418	-1.311779
6	6.208841	-1.052606	0.344869
6	7.107283	-1.382633	1.375681
7	8.150569	-2.154163	1.187820
6	8.332633	-2.620016	-0.069179
7	7.554203	-2.347916	-1.139414
6	6.515047	-1.574268	-0.917369
7	9.383424	-3.419266	-0.269833
1	5.914012	-1.335084	-1.793313
1	9.567385	-3.779970	-1.191788
1	9.994591	-3.636734	0.500692

1	6.944478	-0.996274	2.378057
1	3.907784	-1.028534	-0.862157
1	3.911654	4.692901	-1.081793
1	4.007066	4.829979	0.672306
1	4.777947	2.595792	0.887707
1	4.906970	2.507832	-0.887835
1	-0.956679	-2.832424	-1.575200
1	-2.408377	-2.113857	-0.821866
1	-1.627204	-1.302319	-2.206263
1	-0.559262	3.994181	0.118082
1	-2.727979	2.812197	0.061373
1	-4.252970	1.345012	0.934190
1	-4.374279	1.502814	-0.837973
1	-5.326614	-0.775689	-1.000851
1	-5.095623	-1.004511	0.738487
1	-6.762552	0.745967	1.234700
1	-6.814720	1.223412	-0.467783
1	-9.254475	0.603422	0.925913
1	-9.050468	0.950817	-0.809307
1	-11.168015	-0.310188	-0.309144
1	-10.078584	-1.201456	-1.403132
1	-9.086638	-3.391013	1.242751
1	-8.850854	-3.025819	-0.486117
1	-7.938361	-1.317500	1.885831
1	-6.909979	-2.253911	0.771998
1	1.620634	-0.931373	-0.315220

Copanlisib-prot_N2

Atomic Number	Coordinates (Angstroms) X	Y	Z
6	-7.725048	-1.766447	0.792033
7	-7.725677	-0.562474	-0.037442
6	-9.008811	0.125054	0.101728
6	-10.147712	-0.815453	-0.257049
8	-10.126839	-1.964155	0.564224
6	-8.904406	-2.653581	0.421859
6	-6.629880	0.333407	0.289123
6	-5.262530	-0.268671	-0.030035
6	-4.188951	0.797512	0.018268
8	-2.929500	0.145119	-0.187073
6	-1.801381	0.857214	-0.183666
6	-0.618053	0.121760	-0.365558
6	0.615667	0.793729	-0.298258
6	0.634793	2.197153	-0.075228
6	-0.559984	2.919424	0.065237
6	-1.760360	2.256034	0.009696
7	1.770080	0.058158	-0.425398
6	2.900702	0.658041	-0.365005
7	3.020929	2.025101	-0.194917
6	1.917554	2.790948	-0.055917

7	2.259018	4.075135	0.035108
6	3.715173	4.230388	0.157990
6	4.219377	2.878763	-0.369646
7	4.078765	-0.065560	-0.534901
6	5.064256	-0.090236	0.480179
8	5.006881	0.680032	1.412546
8	-0.601693	-1.208684	-0.519757
6	-1.460782	-1.805238	-1.501522
6	6.126170	-1.083452	0.298536
6	6.976858	-1.374160	1.377115
7	7.956768	-2.244909	1.298137
6	8.117425	-2.854549	0.103127
7	7.381517	-2.637097	-1.006813
6	6.404898	-1.762292	-0.892176
7	9.103687	-3.754685	0.011706
1	5.834932	-1.575361	-1.800294
1	9.263059	-4.231921	-0.859983
1	9.677376	-3.943625	0.817052
1	6.830576	-0.871569	2.328907
1	3.884139	-0.960716	-0.969835
1	4.069380	5.065914	-0.441502
1	3.990272	4.381325	1.203769
1	5.051737	2.493370	0.208435
1	4.462808	2.910631	-1.433275
1	-0.940689	-2.698132	-1.841179
1	-2.420222	-2.069178	-1.061897
1	-1.611858	-1.124145	-2.341755
1	-0.551088	3.991251	0.221531
1	-2.681187	2.807919	0.133953
1	-4.176803	1.300340	0.991845
1	-4.340459	1.544300	-0.768586
1	-5.292036	-0.717601	-1.026324
1	-4.998525	-1.049817	0.686299
1	-6.662993	0.634905	1.353417
1	-6.767829	1.244051	-0.304448
1	-9.153345	0.484886	1.135822
1	-9.021892	0.989619	-0.567584
1	-11.110759	-0.330362	-0.098761
1	-10.062559	-1.109612	-1.312055
1	-8.950941	-3.520589	1.080812
1	-8.788855	-2.997202	-0.615384
1	-7.787477	-1.504408	1.863823
1	-6.801718	-2.326374	0.627740
1	1.600717	4.795850	0.286590

Copanlisib-prot_N3

Atomic Number	Coordinates (Angstroms) X	Y	Z
6	6.375142	-1.676915	-0.855122
6	6.042130	-1.151667	0.393811
6	6.823029	-1.600045	1.466503

7	7.790114	-2.483225	1.329260
6	8.005317	-2.936351	0.077777
7	7.341322	-2.559852	-1.030567
6	4.986356	-0.148846	0.645350
8	4.938839	0.516344	1.653501
7	8.978727	-3.851553	-0.075621
7	4.042195	-0.022081	-0.382299
6	2.880593	0.754345	-0.251018
7	1.748142	0.160357	-0.449415
6	0.602194	0.935792	-0.375397
6	0.625259	2.318468	-0.109131
6	1.928988	2.946711	0.048169
7	3.033728	2.072020	0.012159
6	-0.550442	3.053502	-0.013120
6	-1.775403	2.428269	-0.171557
6	-1.813946	1.056093	-0.437810
6	-0.643546	0.304464	-0.544571
7	2.201725	4.178844	0.188979
6	3.659218	4.293845	0.317674
6	4.252511	2.897232	0.000445
8	-2.978393	0.354262	-0.610594
6	-4.174509	0.962997	-0.208103
6	-5.222936	-0.143483	-0.247070
6	-6.558468	0.367299	0.264505
7	-7.682161	-0.604148	0.013179
6	-7.476621	-1.934314	0.691396
6	-8.675146	-2.835201	0.428676
8	-9.866366	-2.228414	0.860992
6	-10.118762	-1.039024	0.157191
6	-9.014095	-0.024236	0.412307
8	-0.743220	-1.035605	-0.747725
6	-0.365025	-1.460338	-2.054460
1	5.860880	-1.355191	-1.758092
1	9.200206	-4.189278	-0.996534
1	9.522280	-4.130763	0.723260
1	6.634859	-1.215110	2.464524
1	3.839807	-0.877536	-0.885231
1	4.034910	5.061455	-0.358923
1	3.900481	4.602375	1.337792
1	4.956223	2.555274	0.753428
1	4.710762	2.846388	-0.990354
1	-0.473150	-2.543222	-2.068466
1	-1.026757	-1.013170	-2.802940
1	0.671646	-1.190631	-2.260645
1	-0.483929	4.115517	0.188458
1	-2.685518	3.008575	-0.098067
1	-4.079750	1.369030	0.807638
1	-4.451467	1.783555	-0.883051
1	-5.311729	-0.498418	-1.279978
1	-4.848537	-0.968625	0.361108
1	-6.544876	0.545055	1.342084
1	-6.846701	1.295998	-0.233976
1	-8.941304	0.207770	1.476284

1	-9.161731	0.894926	-0.155788
1	-11.065684	-0.637078	0.514920
1	-10.214873	-1.241703	-0.919822
1	-8.554274	-3.761729	0.988208
1	-8.738017	-3.084972	-0.640927
1	-7.369699	-1.720711	1.756833
1	-6.557186	-2.374036	0.307931
1	-7.711313	-0.780564	-0.997473

Copanlisib-prot_N3a

Atomic Number	Coordinates (Angstroms) X	Y	Z
6	-5.322581	-0.962436	-1.139108
6	-4.558489	-0.366380	-0.126019
6	-5.087517	-0.458961	1.163387
7	-6.215419	-1.086195	1.433875
6	-6.849882	-1.642951	0.383577
7	-6.449079	-1.599407	-0.904605
6	-3.316865	0.338529	-0.484789
7	-2.456616	0.610573	0.595888
6	-1.190929	1.171775	0.401641
7	-0.162952	0.498712	0.831877
6	1.078048	1.086904	0.675378
6	1.279677	2.348560	0.086669
6	0.091084	3.082801	-0.324981
7	-1.130701	2.391418	-0.167993
6	2.560320	2.870233	-0.078218
6	3.673534	2.152006	0.325511
6	3.494238	0.893896	0.910977
6	2.215213	0.366377	1.078734
6	-2.218899	3.320349	-0.520541
6	-1.411102	4.549254	-1.020618
7	0.007577	4.262322	-0.778337
8	4.509605	0.086730	1.348372
6	5.768515	0.169472	0.688823
6	5.683617	-0.382709	-0.729198
6	5.176549	-1.821055	-0.783831
7	3.694764	-2.001000	-0.582244
6	3.370285	-3.467745	-0.494828
6	1.866026	-3.678381	-0.408531
8	1.234107	-3.117006	-1.532399
6	1.405070	-1.716599	-1.540432
6	2.880308	-1.358234	-1.676212
8	2.100008	-0.918584	1.553493
6	1.765568	-0.999558	2.947454
7	-7.989967	-2.302176	0.640377
8	-3.037490	0.668399	-1.613947
1	-4.596244	0.014442	2.010883
1	-8.345267	-2.331222	1.581153
1	-8.510729	-2.702990	-0.121629
1	-4.983946	-0.908852	-2.169801

1	-2.437357	-0.096435	1.320794
1	-1.691527	5.464092	-0.498023
1	-1.563109	4.718013	-2.088185
1	-2.847806	2.887452	-1.293771
1	-2.815900	3.531711	0.368612
1	1.678645	-2.058009	3.186147
1	2.557679	-0.543964	3.544930
1	0.812469	-0.498838	3.120322
1	2.663388	3.850985	-0.527172
1	4.661547	2.573361	0.199611
1	6.155503	1.189866	0.693287
1	6.691357	-0.387408	-1.153294
1	5.089669	0.274846	-1.368539
1	5.394761	-2.271443	-1.753970
1	5.662337	-2.422914	-0.011939
1	6.436569	-0.440670	1.298033
1	3.766682	-3.933081	-1.399884
1	3.886044	-3.866556	0.379760
1	1.657293	-4.747708	-0.410794
1	1.470638	-3.235856	0.514373
1	0.873140	-1.326722	-2.407899
1	0.972974	-1.276058	-0.634946
1	3.280782	-1.740808	-2.618306
1	3.020952	-0.279034	-1.618572
1	3.415635	-1.583132	0.323729

Copanlisib-prot_N4

Atomic Number	Coordinates (Angstroms) X	Y	Z
6	-7.722390	-1.837251	0.750398
7	-7.737315	-0.633074	-0.076329
6	-9.030918	0.032282	0.057853
6	-10.152314	-0.923970	-0.313524
8	-10.120079	-2.078203	0.500843
6	-8.882464	-2.744009	0.368472
6	-6.655776	0.280591	0.257849
6	-5.274760	-0.302620	-0.029488
6	-4.215766	0.779521	0.024207
8	-2.950272	0.148487	-0.143095
6	-1.838917	0.909660	-0.106763
6	-0.627695	0.209947	-0.232180
6	0.582420	0.909611	-0.203046
6	0.568412	2.309908	-0.037546
6	-0.636312	2.992325	0.079586
6	-1.836594	2.301773	0.046399
7	1.768944	0.184712	-0.330452
6	2.862388	0.846304	-0.268731
7	2.993653	2.179798	-0.092968
6	1.844088	3.007653	-0.028489
7	2.077418	4.255179	0.007562
6	3.531016	4.436061	0.022771

6	4.157364	3.053608	-0.295563
7	4.055764	0.080344	-0.446015
6	5.049791	0.026330	0.491892
8	5.177596	0.763363	1.436975
8	-0.628109	-1.144758	-0.328613
6	-0.985026	-1.634556	-1.617443
6	6.073446	-1.063656	0.267562
6	6.806203	-1.494613	1.376427
7	7.744549	-2.438767	1.268725
6	7.908592	-2.886974	0.051269
7	7.331073	-2.577060	-1.075104
6	6.382305	-1.633504	-0.961033
7	8.955818	-3.944227	-0.069836
1	5.892356	-1.332515	-1.881093
1	9.062155	-4.202692	-1.057462
1	9.851314	-3.604431	0.299457
1	6.627863	-1.063674	2.355811
1	3.825325	-0.789689	-0.913266
1	3.826412	5.193371	-0.703075
1	3.834541	4.785532	1.013174
1	4.967233	2.791859	0.380290
1	4.492813	2.978656	-1.333946
1	-0.968140	-2.720605	-1.549625
1	-1.986654	-1.300921	-1.896230
1	-0.256703	-1.299149	-2.362189
1	-0.616425	4.068626	0.198364
1	-2.766426	2.843707	0.146496
1	-4.238848	1.300750	0.988866
1	-4.368582	1.515638	-0.773715
1	-5.278187	-0.765711	-1.019781
1	-5.009508	-1.071008	0.699900
1	-6.713614	0.592256	1.318039
1	-6.796959	1.183248	-0.346820
1	-9.188475	0.385257	1.092774
1	-9.053644	0.900401	-0.606634
1	-11.124573	-0.455814	-0.159442
1	-10.054717	-1.210942	-1.369552
1	-8.920102	-3.613800	1.024640
1	-8.751650	-3.082396	-0.668804
1	-7.798000	-1.579009	1.822607
1	-6.787368	-2.379042	0.592961
1	8.693588	-4.773870	0.475007

Copanlisib-prot_N5

Atomic Number	Coordinates (Angstroms) X	Y	Z
6	6.405181	-1.604121	-0.944264
6	6.076538	-1.077871	0.264906
6	6.846286	-1.516391	1.375510
7	7.797769	-2.414176	1.295969
6	8.080029	-2.930550	0.093155

7	7.408143	-2.526066	-1.017541
6	5.037590	-0.009811	0.500705
8	5.160445	0.717175	1.452878
7	9.037050	-3.843789	0.001221
7	4.045009	0.043846	-0.441120
6	2.855103	0.818483	-0.266292
7	1.759686	0.161482	-0.332896
6	0.575077	0.890837	-0.208714
6	0.567053	2.291147	-0.042864
6	1.845481	2.983732	-0.029604
7	2.992701	2.150866	-0.089490
6	-0.634990	2.978518	0.072342
6	-1.837903	2.292622	0.037770
6	-1.846057	0.900555	-0.115187
6	-0.637375	0.195786	-0.240826
7	2.084258	4.229905	0.007735
6	3.538057	4.405588	0.029781
6	4.161259	3.019980	-0.282979
8	-2.960142	0.144147	-0.150210
6	-4.223482	0.779467	0.021213
6	-5.286195	-0.299021	-0.030429
6	-6.664595	0.288936	0.260179
7	-7.749721	-0.620683	-0.072978
6	-7.738021	-1.825248	0.753392
6	-8.901940	-2.727663	0.372819
8	-10.136825	-2.057354	0.507206
6	-10.166140	-0.902902	-0.306875
6	-9.040842	0.049293	0.063174
8	-0.641283	-1.158707	-0.337422
6	-1.018534	-1.650372	-1.619873
1	5.938706	-1.330299	-1.882982
1	9.512393	-4.103382	0.855635
1	6.643605	-1.087279	2.353261
1	3.793482	-0.837938	-0.875454
1	3.839831	5.160459	-0.695963
1	3.838112	4.755643	1.020938
1	4.964751	2.756543	0.399973
1	4.505245	2.943902	-1.318589
1	-1.000389	-2.736269	-1.550339
1	-2.024190	-1.317483	-1.883874
1	-0.301838	-1.315951	-2.376359
1	-0.611116	4.054664	0.191699
1	-2.765642	2.838155	0.137652
1	-4.241626	1.299889	0.986345
1	-4.375744	1.516578	-0.775831
1	-5.293635	-0.761628	-1.020939
1	-5.022108	-1.068638	0.698076
1	-6.719227	0.599783	1.320764
1	-6.803714	1.192666	-0.343378
1	-9.195670	0.402511	1.098393
1	-9.061406	0.917649	-0.601074
1	-11.136486	-0.431296	-0.151347
1	-10.071046	-1.189980	-1.363105

1	-8.941748	-3.597493	1.028785
1	-8.773856	-3.066222	-0.664747
1	-7.811203	-1.567052	1.825749
1	-6.805225	-2.370466	0.594490
1	9.310479	-4.290871	-0.860487
1	7.657950	-2.903934	-1.925440

Copanlisib-prot_O6

Atomic Number	Coordinates (Angstroms) X	Y	Z
6	-9.039669	0.142111	0.181485
7	-7.766806	-0.547441	-0.020861
6	-7.768622	-1.802774	0.727328
6	-8.961905	-2.651920	0.315639
8	-10.176000	-1.962021	0.518376
6	-10.193662	-0.760163	-0.223730
6	-6.656098	0.314331	0.348481
6	-5.298175	-0.279231	-0.020220
6	-4.213309	0.771740	0.086510
8	-2.963489	0.128631	-0.170704
6	-1.837648	0.854102	-0.115895
6	-1.787709	2.225484	0.154056
6	-0.568530	2.885548	0.213412
6	0.612269	2.185913	0.014188
6	0.575257	0.805635	-0.278762
6	-0.645255	0.133539	-0.342167
6	1.904593	2.852383	0.003643
7	3.058643	1.989819	-0.037588
6	2.849544	0.671465	-0.380252
7	1.746715	0.063242	-0.489639
6	4.157830	2.856336	-0.532630
6	3.627121	4.231386	-0.081028
7	2.170524	4.089685	-0.027956
7	4.042864	-0.116244	-0.518505
6	5.004743	-0.184669	0.397781
8	4.963910	0.579677	1.454902
8	-0.669422	-1.201252	-0.546617
6	-1.174957	-1.612958	-1.819204
6	6.113019	-1.076499	0.287267
6	6.432094	-1.781487	-0.892464
7	7.443431	-2.602225	-0.973851
6	8.189679	-2.751084	0.151114
7	7.995825	-2.107461	1.328901
6	6.979800	-1.289156	1.384747
7	9.206557	-3.599647	0.088519
1	5.860536	-1.648827	-1.809641
1	9.789806	-3.738569	0.899900
1	6.811875	-0.774157	2.325997
1	3.939693	-0.895489	-1.159223
1	3.911628	5.027314	-0.766884
1	3.999836	4.491666	0.915187

1	5.120360	2.599251	-0.092015
1	4.216228	2.780645	-1.622225
1	-1.141017	-2.699791	-1.819141
1	-2.202490	-1.275355	-1.957305
1	-0.536474	-1.222644	-2.616650
1	-0.522402	3.948323	0.416084
1	-2.699376	2.779678	0.327800
1	-4.191626	1.212892	1.089932
1	-4.367667	1.571390	-0.646723
1	-5.340235	-0.664115	-1.042522
1	-5.035230	-1.107376	0.641372
1	-6.676789	0.549620	1.429640
1	-6.789888	1.261561	-0.185637
1	-9.166883	0.433924	1.239209
1	-9.051752	1.049678	-0.428234
1	-11.149374	-0.277057	-0.020730
1	-10.124911	-0.984146	-1.297037
1	-9.009812	-3.559935	0.916898
1	-8.862954	-2.927689	-0.743417
1	-7.814242	-1.611575	1.814999
1	-6.853226	-2.359037	0.514065
1	9.390413	-4.098252	-0.768814
1	4.169247	1.149487	1.478512

Olaparib-neut

Atomic Number	Coordinates (Angstroms) X	Y	Z
6	2.117447	0.387506	0.591384
7	1.975193	0.789277	-0.806105
6	3.027879	0.283669	-1.679349
6	4.394540	0.699002	-1.131060
7	4.536129	0.275544	0.258652
6	3.485009	0.824156	1.111693
6	0.887318	1.388214	-1.365449
8	0.722352	1.476837	-2.568188
6	5.088577	-0.929274	0.631458
6	6.119313	-1.524830	-0.275641
6	5.866021	-2.954948	-0.713876
6	6.929594	-2.659779	0.288616
6	-0.155661	1.967230	-0.442356
6	-1.492469	1.652446	-0.676887
6	-2.515314	2.203339	0.090368
6	-2.183459	3.116111	1.091607
6	-0.860857	3.471653	1.326089
6	0.127494	2.886695	0.557081
6	-3.963194	1.840135	-0.179265
9	1.409240	3.231253	0.790891
8	4.769968	-1.485050	1.668634
1	-1.722234	0.966548	-1.485853
1	-0.585509	4.189994	2.087758
1	-2.968070	3.562444	1.693840

1	2.971722	-0.812102	-1.723409
1	2.863668	0.682560	-2.678454
1	5.179053	0.269238	-1.748251
1	4.485462	1.788569	-1.168000
1	3.640424	0.466534	2.126633
1	3.554202	1.914296	1.085647
1	2.050830	-0.705841	0.653767
1	1.315770	0.809671	1.191871
1	7.961733	-2.721771	-0.028013
1	6.723482	-2.912763	1.319316
1	6.154115	-3.224695	-1.720966
1	4.954442	-3.407623	-0.345637
6	-4.163086	0.377915	-0.482647
7	-4.532521	0.086128	-1.683051
7	-4.708262	-1.213176	-2.000788
6	-4.542312	-2.323566	-1.198567
6	-4.118201	-1.989044	0.171459
6	-3.929505	-0.646566	0.528703
1	-4.997555	-1.379813	-2.955060
8	-4.738199	-3.444356	-1.621533
6	-3.916139	-3.015716	1.096828
6	-3.528992	-0.347186	1.841491
1	-4.575292	2.127730	0.680895
1	-4.331033	2.399286	-1.042282
6	-3.522946	-2.704585	2.384323
6	-3.330694	-1.367032	2.753421
1	-4.075881	-4.037531	0.774612
1	-3.362395	-3.492517	3.109970
1	-3.021439	-1.128610	3.764130
1	-3.366133	0.681461	2.138156
1	6.618572	-0.873466	-0.977519

Olaparib-prot_N1

Atomic Number	Coordinates (Angstroms) X	Y	Z
6	3.553400	1.265375	2.845651
6	3.670922	0.261764	1.901366
6	4.000994	0.593435	0.578438
6	4.199770	1.943905	0.228330
6	4.078646	2.945337	1.184255
6	3.757993	2.602446	2.490016
6	4.142643	-0.423923	-0.433435
7	4.438662	-0.049787	-1.646757
7	4.633360	1.238165	-2.031758
6	4.534895	2.328728	-1.159017
6	3.929836	-1.887360	-0.194050
6	2.467098	-2.206764	0.071297
6	1.461376	-1.535129	-0.611051
6	0.116318	-1.842811	-0.419889
6	-0.182509	-2.885713	0.446301
6	0.797326	-3.578395	1.141430

6	2.126609	-3.227935	0.959655
6	-0.876761	-1.122475	-1.307836
8	-0.567489	-0.967151	-2.477673
9	-1.454636	-3.258178	0.616054
8	4.717305	3.439750	-1.565665
7	-2.042757	-0.698333	-0.770178
6	-2.307093	-0.495561	0.657245
6	-3.701269	-1.014786	0.996318
7	-4.687765	-0.351965	0.151152
6	-4.437651	-0.587226	-1.266640
6	-3.041467	-0.093132	-1.653804
6	-5.198197	0.845630	0.622082
8	-4.874799	1.282742	1.711624
6	-6.178669	1.559988	-0.246648
6	-6.996629	2.636387	0.417392
6	-5.893730	3.029787	-0.502225
1	1.681769	-0.762911	-1.341961
1	0.505028	-4.385737	1.800824
1	2.898801	-3.769551	1.495473
1	-2.998746	0.999077	-1.553915
1	-2.795996	-0.359076	-2.679970
1	-5.179612	-0.082026	-1.879635
1	-4.512799	-1.661660	-1.455843
1	-3.937928	-0.810117	2.037664
1	-3.752514	-2.089910	0.808791
1	-2.276835	0.580085	0.868283
1	-1.544352	-0.981050	1.260335
1	-8.015166	2.748088	0.072475
1	-6.828843	2.763610	1.477767
1	-6.140370	3.418888	-1.480763
1	-4.992502	3.422240	-0.049055
1	4.845481	1.402551	-3.008864
1	4.545782	-2.188705	0.657000
1	4.292470	-2.454308	-1.057896
1	4.237924	3.974413	0.885835
1	3.662285	3.377438	3.240687
1	3.298622	1.013196	3.867295
1	3.494177	-0.769682	2.179824
1	-6.659346	1.000871	-1.035840
1	4.530654	-0.740833	-2.387589

Olaparib-prot_N2

Atomic Number	Coordinates (Angstroms) X	Y	Z
6	0.944070	1.652575	2.737010
6	1.989052	1.523214	1.838043
6	1.891847	2.099096	0.559354
6	0.731351	2.815671	0.229947
6	-0.324814	2.936837	1.138378
6	-0.222263	2.347236	2.385389

6	2.915412	1.937247	-0.469910
7	2.820052	2.494026	-1.628798
7	1.741915	3.258322	-1.887809
6	0.618713	3.444798	-1.100015
6	4.060308	0.974752	-0.284039
6	3.568480	-0.458709	-0.160168
6	2.307631	-0.816131	-0.605195
6	1.785942	-2.104884	-0.417183
6	2.607175	-3.062887	0.188691
6	3.887686	-2.744835	0.609093
6	4.353341	-1.449024	0.444704
6	0.412937	-2.468213	-0.786515
8	-0.066489	-3.540909	-0.916146
9	2.149383	-4.285923	0.402708
8	-0.344399	4.055307	-1.505419
7	-0.533912	-1.255313	-1.006593
6	-0.731963	-0.426207	0.260861
6	-1.638922	-1.177416	1.218855
7	-2.928337	-1.414006	0.591136
6	-2.803944	-2.283497	-0.567676
6	-1.861784	-1.669322	-1.608641
6	-3.805252	-0.325510	0.603528
8	-3.489034	0.702386	1.169610
6	-5.110137	-0.496882	-0.082011
6	-6.179929	0.511249	0.258018
6	-5.499996	0.605869	-1.059167
1	1.714844	-0.051408	-1.093171
1	4.488032	-3.513849	1.078508
1	5.346314	-1.196973	0.802126
1	-2.296901	-0.750492	-2.008731
1	-1.650205	-2.363818	-2.420145
1	-3.767211	-2.429400	-1.051318
1	-2.439575	-3.259671	-0.247510
1	-1.771238	-0.566184	2.109448
1	-1.205429	-2.138890	1.511836
1	-1.207763	0.507151	-0.046704
1	0.245954	-0.211420	0.686823
1	-7.193606	0.137010	0.286530
1	-5.914068	1.241133	1.009908
1	-6.036673	0.304592	-1.948406
1	-4.781709	1.406016	-1.186008
1	1.736157	3.688182	-2.804370
1	4.644061	1.227252	0.605981
1	4.722680	1.074413	-1.146478
1	-1.205560	3.494050	0.840967
1	-1.037716	2.432824	3.093225
1	1.030508	1.214509	3.724513
1	2.879776	0.976018	2.123998
1	-5.438234	-1.502742	-0.300842
1	-0.075182	-0.650643	-1.692563

Olaparib-prot_N3

Atomic Number	Coordinates (Angstroms) X	Y	Z
6	4.001965	1.068817	2.809155
6	3.983477	0.126328	1.797424
6	4.156368	0.525324	0.461858
6	4.337494	1.887505	0.183426
6	4.354291	2.835666	1.208710
6	4.188057	2.426234	2.518346
6	4.164556	-0.412324	-0.654036
7	4.336375	-0.031509	-1.873842
7	4.512339	1.281973	-2.115983
6	4.524000	2.326813	-1.210772
6	3.925404	-1.885438	-0.443104
6	2.485512	-2.187998	-0.075385
6	1.446693	-1.598105	-0.790932
6	0.113205	-1.853314	-0.475872
6	-0.152883	-2.750460	0.549741
6	0.846825	-3.372559	1.268074
6	2.168639	-3.076684	0.952054
6	-0.946376	-1.245879	-1.344843
8	-0.855065	-1.274953	-2.549690
9	-1.444338	-3.038452	0.851649
8	4.672444	3.473790	-1.572288
7	-2.036366	-0.657694	-0.725433
6	-2.139291	-0.262544	0.664606
6	-3.444914	-0.786300	1.256174
7	-4.639504	-0.404059	0.403927
6	-4.421902	-0.748899	-1.063360
6	-3.112875	-0.150607	-1.548218
6	-5.060902	1.072229	0.575276
8	-4.306587	1.793519	1.133907
6	-6.368977	1.376280	0.006764
6	-7.104760	2.556630	0.636767
6	-6.488777	2.738230	-0.688909
1	1.664871	-0.931338	-1.618841
1	0.587112	-4.077648	2.047345
1	2.964779	-3.557817	1.510241
1	-3.146740	0.946635	-1.514529
1	-2.940155	-0.451665	-2.581098
1	-5.280002	-0.376466	-1.621740
1	-4.385606	-1.837679	-1.119199
1	-3.622388	-0.385177	2.252838
1	-3.431990	-1.876146	1.279230
1	-2.095458	0.826549	0.746986
1	-1.317295	-0.672827	1.246753
1	-8.177903	2.443276	0.703461
1	-6.615297	3.007660	1.489947
1	-7.120213	2.753096	-1.566519
1	-5.573081	3.312279	-0.747951
1	4.655366	1.519068	-3.088812
1	4.592849	-2.274950	0.329737
1	4.176588	-2.391374	-1.377647

1	4.506026	3.875683	0.945993
1	4.207941	3.151949	3.322084
1	3.878920	0.752956	3.838299
1	3.840389	-0.919756	2.039111
1	-6.957298	0.555424	-0.380991
1	-5.437605	-0.959150	0.726681

Olaparib-prot_N4

Atomic Number	Coordinates (Angstroms) X	Y	Z
6	4.205418	1.172580	2.795358
6	4.139697	0.181149	1.833579
6	4.315399	0.503833	0.477964
6	4.546879	1.841773	0.128400
6	4.611267	2.839752	1.103360
6	4.442390	2.504811	2.433684
6	4.278412	-0.489641	-0.587956
7	4.455137	-0.178769	-1.826713
7	4.678430	1.112977	-2.137672
6	4.736749	2.201630	-1.287993
6	3.981129	-1.939486	-0.300830
6	2.526594	-2.160966	0.066933
6	1.521669	-1.619796	-0.730967
6	0.174376	-1.796362	-0.420895
6	-0.140834	-2.565772	0.691625
6	0.824144	-3.138363	1.494060
6	2.161062	-2.922860	1.176408
6	-0.845270	-1.260303	-1.381558
8	-0.716464	-1.418095	-2.572381
9	-1.445382	-2.775317	0.998554
8	4.923913	3.322439	-1.708982
7	-1.941833	-0.591355	-0.862810
6	-2.057991	-0.042208	0.469270
6	-3.407599	-0.455821	1.055403
7	-4.498400	0.000176	0.162810
6	-4.353134	-0.558668	-1.203821
6	-2.988898	-0.161361	-1.766633
6	-5.439619	0.839949	0.510985
8	-5.423798	1.450995	1.677089
6	-6.591427	1.185088	-0.310142
6	-7.834299	1.727635	0.389633
6	-6.990808	2.661950	-0.380898
1	1.778571	-1.055624	-1.621759
1	0.525788	-3.746273	2.338651
1	2.929710	-3.368232	1.799159
1	-2.951670	0.925995	-1.911290
1	-2.834609	-0.641416	-2.731851
1	-5.141423	-0.184621	-1.847195
1	-4.432515	-1.644593	-1.122011
1	-3.534132	-0.084812	2.071780
1	-3.468517	-1.544957	1.096066

1	-1.996148	1.054144	0.426655
1	-1.255415	-0.397864	1.110422
1	-8.767936	1.393296	-0.040964
1	-7.804963	1.783018	1.468261
1	-7.323345	3.000032	-1.352419
1	-6.368156	3.352660	0.171498
1	4.822516	1.293849	-3.122353
1	4.626219	-2.313023	0.498171
1	4.218532	-2.503907	-1.204797
1	4.801741	3.857986	0.786114
1	4.500360	3.269159	3.198872
1	4.080951	0.914652	3.840367
1	3.958475	-0.844597	2.130115
1	-6.764584	0.586483	-1.187850
1	-4.605410	1.341333	2.174438

Olaparib-prot_N4a

Atomic Number	Coordinates (Angstroms) X	Y	Z
6	1.590534	2.713825	-0.410871
7	0.324544	2.094882	-0.047156
6	0.386290	0.660038	0.170032
6	1.501797	0.316323	1.149117
7	2.778251	0.878581	0.685714
6	2.700654	2.348211	0.571914
6	-0.839992	2.828946	-0.265776
8	-0.807918	4.015702	-0.473819
6	3.823367	0.146389	0.358049
6	5.148056	0.719552	0.128298
6	5.927583	0.208559	-1.082969
6	6.349287	-0.205818	0.270988
6	-2.124843	2.049143	-0.259871
6	-2.277982	0.941285	-1.086396
6	-3.422585	0.150980	-1.059435
6	-4.480605	0.550623	-0.244400
6	-4.380245	1.691773	0.544674
6	-3.198090	2.414244	0.543765
6	-3.403540	-1.180911	-1.798518
6	-2.103942	-1.863356	-1.438550
6	-1.829779	-2.281558	-0.068804
6	-0.503171	-2.582507	0.281350
6	0.530752	-2.453310	-0.736266
7	0.062491	-2.249989	-1.991072
7	-1.195074	-1.886754	-2.354596
6	-0.167099	-2.969047	1.585097
6	-1.158937	-3.055560	2.541130
6	-2.485767	-2.748074	2.205519
6	-2.821486	-2.363138	0.921548
8	1.751889	-2.496584	-0.515258
9	-3.076559	3.456159	1.364487
8	3.761471	-1.143252	0.249431

1	-1.459343	0.661045	-1.740933
1	-5.191686	2.014738	1.184544
1	-5.390734	-0.039194	-0.209294
1	1.888277	2.390846	-1.418000
1	1.450871	3.793717	-0.423023
1	3.641301	2.753052	0.213673
1	2.488355	2.749454	1.565478
1	1.582950	-0.759676	1.282780
1	1.294844	0.771688	2.121281
1	0.573442	0.134773	-0.778098
1	-0.552789	0.300624	0.586657
1	7.223622	0.257561	0.706381
1	6.145836	-1.221505	0.577520
1	6.503920	0.953603	-1.613506
1	5.422914	-0.526192	-1.695790
1	0.739039	-2.214066	-2.744329
1	-4.269070	-1.784534	-1.518696
1	-3.423942	-1.042844	-2.880492
1	0.862822	-3.219521	1.811486
1	-0.917699	-3.368773	3.549298
1	-3.257782	-2.817704	2.962132
1	-3.849886	-2.126723	0.681583
1	5.300722	1.745242	0.420200
1	2.886487	-1.594683	0.027962

Olaparib-prot_N5

Atomic Number	Coordinates (Angstroms) X	Y	Z
6	3.597026	1.344269	2.806555
6	3.709454	0.326626	1.876441
6	3.980960	0.630802	0.532599
6	4.124526	1.975327	0.159585
6	4.010974	2.998761	1.102639
6	3.748442	2.682547	2.422794
6	4.122537	-0.385914	-0.501007
7	4.367408	-0.094501	-1.733580
7	4.504607	1.201589	-2.068016
6	4.410034	2.314490	-1.246095
6	3.938101	-1.848519	-0.190918
6	2.489820	-2.211722	0.074747
6	1.467592	-1.596056	-0.639661
6	0.128263	-1.923892	-0.400765
6	-0.168936	-2.901489	0.544452
6	0.824810	-3.541418	1.255732
6	2.147219	-3.184950	1.017750
6	-0.923321	-1.290120	-1.216383
8	-0.781267	-1.315957	-2.527069
9	-1.451639	-3.229690	0.747222
8	4.546514	3.432209	-1.687182
7	-1.966395	-0.682420	-0.747334

6	-2.188349	-0.404369	0.692674
6	-3.562485	-0.934862	1.098247
7	-4.590232	-0.340510	0.257715
6	-4.394895	-0.659879	-1.144854
6	-3.030730	-0.149575	-1.631868
6	-5.039129	0.924650	0.649830
8	-4.600854	1.444517	1.654914
6	-6.070280	1.570605	-0.202955
6	-6.813124	2.729459	0.413314
6	-5.785857	3.006325	-0.624115
1	1.713743	-0.827497	-1.367379
1	0.555775	-4.304561	1.975006
1	2.932522	-3.680806	1.578242
1	-2.989974	0.941156	-1.559174
1	-2.830481	-0.450562	-2.656141
1	-5.166071	-0.205984	-1.762655
1	-4.452210	-1.744132	-1.274464
1	-3.751480	-0.670278	2.135990
1	-3.598844	-2.020138	0.985884
1	-2.175307	0.682829	0.807669
1	-1.380554	-0.833838	1.277613
1	-7.854957	2.825902	0.141763
1	-6.559058	2.955626	1.439558
1	-6.106878	3.300438	-1.614082
1	-4.845788	3.423570	-0.286248
1	4.730449	1.370746	-3.039639
1	4.549117	-2.140206	0.666735
1	4.298916	-2.414234	-1.053064
1	4.138817	4.022369	0.771514
1	3.664549	3.467891	3.163728
1	3.397440	1.103566	3.843838
1	3.595831	-0.703770	2.192253
1	-6.623050	0.944985	-0.888890
1	-0.036529	-1.877692	-2.782258

1. Geometries of neutral and protonated species together with one water molecule optimized utilizing SMD-M06-2X/6-311+G(d,p) model.

Palbociclib-neut

Atomic Number	Coordinates (Angstroms) X	Y	Z
6	7.470473	0.575655	1.175164
7	6.923415	0.207325	-0.145050
6	7.506705	-1.055665	-0.603624
6	9.020909	-0.963509	-0.629094
7	9.527590	-0.650934	0.708980
6	8.984092	0.642658	1.128643
6	5.514652	0.269075	-0.206889

6	4.854096	1.460913	0.120299
7	3.535437	1.618425	0.088557
6	2.775274	0.589366	-0.297366
6	3.323016	-0.627544	-0.689744
6	4.701273	-0.786465	-0.627495
7	1.404320	0.894377	-0.331667
6	0.316209	0.081651	-0.248112
7	0.495872	-1.245066	-0.112671
6	-0.609406	-1.966019	-0.047029
6	-1.899295	-1.430359	-0.103309
6	-1.956568	-0.023997	-0.220930
7	-0.858593	0.718181	-0.296242
7	-3.182036	0.612815	-0.254818
6	-4.356934	-0.099848	-0.081794
6	-4.286491	-1.554629	-0.024279
6	-3.097010	-2.220556	-0.024160
6	-3.251891	2.090868	-0.361111
6	-4.230705	2.624532	-1.441693
6	-5.156543	3.602653	-0.700912
6	-4.317374	4.068643	0.495784
6	-3.630318	2.781994	0.962161
8	-5.431552	0.498569	0.030089
6	-5.605431	-2.262279	0.034835
6	-6.659385	-1.928702	-0.985463
6	-2.972865	-3.715324	0.020746
8	-5.810158	-3.111726	0.883856
1	-0.472864	-3.035791	0.068665
1	-2.287272	-4.055525	-0.757289
1	-2.559019	-4.024326	0.984727
1	-3.929295	-4.210628	-0.116334
1	1.196726	1.884752	-0.285903
1	5.426282	2.335475	0.409992
1	5.124418	-1.737817	-0.921189
1	2.694795	-1.433695	-1.033179
1	7.202027	-1.886722	0.051383
1	7.147302	-1.267433	-1.611914
1	9.425911	-1.921936	-0.955996
1	9.317867	-0.200329	-1.362867
1	9.356730	0.888039	2.123902
1	9.282625	1.449268	0.443492
1	7.155849	-0.166911	1.923918
1	7.081182	1.546733	1.475651
1	-4.335087	2.166105	1.523941
1	-2.751990	2.955904	1.586055
1	-3.565694	4.791569	0.162220
1	-4.910857	4.537567	1.282084
1	-5.484918	4.426444	-1.336439
1	-6.044422	3.078286	-0.339164
1	-3.648059	3.157402	-2.195883
1	-4.773177	1.827351	-1.947881
1	-2.247652	2.390208	-0.633956
1	10.538280	-0.569944	0.655622
1	-7.050716	-2.868006	-1.381275

1	-7.477614	-1.404305	-0.484857
1	-6.278269	-1.307445	-1.793664
1	-6.710014	0.059445	1.331949
8	-7.325359	-0.171933	2.047329
1	-7.427458	-1.127568	1.976596

Palbociclib-prot_N2

Atomic Number	Coordinates (Angstroms) X	Y	Z
6	-4.885075	2.530071	-1.111581
6	-3.925280	2.036667	0.004708
6	-4.547813	2.457907	1.348853
6	-5.422855	3.653595	0.961602
6	-6.043778	3.217399	-0.371575
7	-3.591004	0.589676	-0.055328
6	-4.645576	-0.332240	-0.033713
6	-4.325467	-1.759083	-0.072996
6	-3.051826	-2.214829	-0.138584
6	-1.999884	-1.222518	-0.155606
6	-2.291021	0.174543	-0.104529
6	-0.671522	-1.552033	-0.247227
7	0.275253	-0.603177	-0.259057
6	-0.069503	0.711566	-0.172146
7	-1.334296	1.097651	-0.097706
8	-5.801335	0.063037	-0.003339
6	-2.666661	-3.662034	-0.159525
6	-5.509900	-2.682148	-0.025802
6	-6.494689	-2.540163	1.099455
7	0.893892	1.637437	-0.189649
6	2.287490	1.349584	-0.086912
7	2.680461	0.719228	1.017416
6	3.964721	0.406491	1.131143
6	4.940441	0.716612	0.163520
6	4.500792	1.416337	-0.965987
6	3.153618	1.731955	-1.094925
7	6.274896	0.349375	0.378476
6	7.216647	0.757789	-0.667983
6	8.642353	0.445592	-0.254472
7	8.788237	-0.987621	0.005455
6	7.897442	-1.356121	1.107110
6	6.458237	-1.074919	0.723996
8	-5.639390	-3.542920	-0.874642
1	-0.312243	-2.570041	-0.325529
1	-6.690527	-3.535515	1.502861
1	-6.136152	-1.876718	1.884065
1	-7.431123	-2.145934	0.697450
1	-2.217667	-3.911204	-1.125036
1	-1.920963	-3.857877	0.613582
1	-3.519771	-4.313463	0.003366
1	0.586881	2.603280	-0.224472
1	4.248452	-0.098801	2.046951

1	5.182838	1.698280	-1.755995
1	2.781700	2.247044	-1.971602
1	6.987293	0.246264	-1.615129
1	7.124732	1.832898	-0.828232
1	9.312574	0.739297	-1.062969
1	8.894335	1.045356	0.631944
1	9.745114	-1.162716	0.296413
1	8.000986	-2.420760	1.319696
1	8.135561	-0.799152	2.024786
1	6.175302	-1.696526	-0.138648
1	5.814467	-1.337738	1.560291
1	-5.170191	1.656927	1.751266
1	-3.778372	2.698672	2.083929
1	-4.796394	4.538127	0.807297
1	-6.164758	3.894414	1.724351
1	-6.461133	4.050958	-0.938260
1	-6.845989	2.500419	-0.181925
1	-4.348094	3.254743	-1.726706
1	-5.216968	1.724689	-1.765249
1	-2.975561	2.542749	-0.110601
1	1.260354	-0.906866	-0.392898
8	2.718402	-1.804460	-0.887999
1	3.257198	-2.044590	-0.123666
1	3.279289	-1.199577	-1.391695

Palbociclib-prot_N3

Atomic Number	Coordinates (Angstroms) X	Y	Z
6	-7.210383	1.090361	0.750424
7	-6.439150	0.337022	-0.259670
6	-6.843712	-1.072015	-0.282552
6	-8.340197	-1.192380	-0.498995
7	-9.059909	-0.488013	0.563150
6	-8.698610	0.929777	0.513869
6	-5.058274	0.558528	-0.201540
6	-4.131765	-0.460846	-0.155321
7	-2.798791	-0.195926	-0.162074
6	-2.292962	1.039385	-0.182829
6	-3.191938	2.117395	-0.220049
6	-4.538510	1.877327	-0.235628
7	-0.944245	1.280251	-0.171849
6	0.107651	0.389605	-0.108343
7	1.308718	0.948582	-0.061838
6	2.352676	0.123254	0.004379
6	2.183207	-1.281095	0.012521
6	0.867752	-1.730266	-0.040563
7	-0.178303	-0.914929	-0.099594
6	3.322429	-2.163045	0.076706
6	4.545850	-1.587747	0.129700
6	4.742450	-0.143814	0.131223
7	3.615834	0.668881	0.069905

6	3.805227	2.140548	0.063704
6	4.503158	2.662071	-1.205815
6	5.192369	3.938852	-0.716294
6	5.733064	3.537815	0.662169
6	4.600452	2.698041	1.275263
6	3.108080	-3.646073	0.059309
6	5.819120	-2.391236	0.192602
8	6.116672	-2.982246	1.210949
8	5.868543	0.344484	0.199884
6	6.632517	-2.464756	-1.059525
1	0.649228	-2.792832	-0.032653
1	6.101932	-3.136671	-1.744298
1	6.694642	-1.488865	-1.542357
1	7.624041	-2.864561	-0.852436
1	2.550858	-3.954613	0.947597
1	2.517757	-3.928078	-0.815481
1	4.049525	-4.191115	0.035031
1	-0.681802	2.260061	-0.179148
1	-4.371734	-1.511718	-0.119145
1	-5.215568	2.720407	-0.293388
1	-2.797929	3.124946	-0.250112
1	-6.952118	0.723509	1.754071
1	-6.955743	2.147053	0.695772
1	-9.236308	1.470658	1.293284
1	-8.955210	1.380891	-0.455413
1	-10.055581	-0.570479	0.382006
1	-8.610552	-2.248623	-0.484539
1	-8.588003	-0.790528	-1.491886
1	-6.564647	-1.568437	0.659442
1	-6.327930	-1.574802	-1.102503
1	5.249858	1.946710	-1.554626
1	3.786747	2.832912	-2.010923
1	4.454792	4.740629	-0.606640
1	5.969052	4.285952	-1.399355
1	5.996151	4.396265	1.281885
1	6.628609	2.924493	0.535655
1	3.933454	3.332913	1.861776
1	4.966737	1.909817	1.931593
1	2.802469	2.547371	0.095512
1	-2.155955	-1.001253	-0.136421
8	-2.434039	-3.023844	-0.011690
1	-3.095201	-3.585713	-0.431291
1	-1.607952	-3.512644	-0.096850

Palbociclib-prot_N4

Atomic Number	Coordinates (Angstroms) X	Y	Z
6	6.810491	-0.516088	1.342898
7	6.297617	0.013830	0.027997
6	6.872433	-0.771311	-1.123195
6	8.385315	-0.765404	-1.038285

7	8.814790	-1.347249	0.233501
6	8.325759	-0.513485	1.331965
6	4.840620	0.096650	-0.018255
6	4.239495	1.333774	-0.161659
7	2.919683	1.489181	-0.207615
6	2.144190	0.403202	-0.110856
6	2.669094	-0.888954	0.035696
6	4.042557	-1.035093	0.081190
7	0.789821	0.712772	-0.150961
6	-0.334497	-0.072283	-0.145712
7	-0.212851	-1.405649	-0.187891
6	-1.353245	-2.081622	-0.171086
6	-2.611077	-1.484534	-0.122023
6	-2.608240	-0.071999	-0.102886
7	-1.473885	0.620236	-0.107264
7	-3.801811	0.622051	-0.077219
6	-5.016564	-0.049171	-0.026968
6	-4.995352	-1.506030	-0.032686
6	-3.847387	-2.223385	-0.089067
6	-3.811384	2.104888	-0.085573
6	-4.646823	2.743407	-1.228494
6	-5.611132	3.715565	-0.529706
6	-4.896235	4.065909	0.781628
6	-4.314701	2.722796	1.232005
8	-6.079260	0.571686	0.000631
6	-6.354466	-2.151051	0.024206
8	-6.796793	-2.731645	-0.947311
6	-3.809889	-3.722108	-0.086890
6	-7.076467	-2.096881	1.332971
1	-1.268103	-3.162635	-0.202549
1	-8.129288	-2.343421	1.202918
1	-6.605624	-2.844076	1.982814
1	-6.955258	-1.124284	1.810131
1	-3.367067	-4.086902	-1.017292
1	-3.186794	-4.079127	0.736261
1	-4.803336	-4.153490	0.015541
1	0.601696	1.708204	-0.178729
1	4.841860	2.233295	-0.240956
1	4.477989	-2.021491	0.195989
1	2.020236	-1.744369	0.112719
1	6.475848	-1.784348	-1.049461
1	6.518666	-0.297548	-2.038097
1	8.768325	-1.360635	-1.866820
1	8.748649	0.264796	-1.163085
1	9.829680	-1.342702	0.258503
1	8.667708	-0.917143	2.284568
1	8.683280	0.523341	1.253407
1	6.417309	-1.526569	1.458563
1	6.412017	0.131855	2.122748
1	-5.101079	2.104649	1.668585
1	-3.507516	2.818259	1.960119
1	-4.085180	4.774478	0.583967
1	-5.557433	4.509614	1.527521

1	-5.832994	4.592105	-1.140259
1	-6.554632	3.211877	-0.305259
1	-3.968090	3.291734	-1.884830
1	-5.161868	1.999205	-1.834410
1	-2.774427	2.384837	-0.222354
1	6.670956	0.987036	-0.057553
8	7.453309	2.574773	-0.074230
1	8.386779	2.431396	-0.273042
1	7.131135	3.146591	-0.781527

Palbociclib-prot_N5

Atomic Number	Coordinates (Angstroms) X	Y	Z
6	-4.921850	2.625217	-1.382066
6	-4.089799	2.082405	-0.188333
6	-4.653193	2.743063	1.083271
6	-5.271018	4.038866	0.550766
6	-5.938357	3.595201	-0.757376
7	-4.013536	0.604429	-0.101569
6	-5.197140	-0.118785	-0.037061
6	-5.110130	-1.569931	0.050346
6	-3.927775	-2.233998	0.059619
6	-2.727533	-1.442471	0.001936
6	-2.787485	-0.032967	-0.070016
6	-1.441272	-1.981976	0.022439
7	-0.333468	-1.259467	-0.024525
6	-0.513791	0.070262	-0.093175
7	-1.687192	0.709750	-0.113203
8	-6.287868	0.452866	-0.073095
6	-3.819496	-3.726513	0.156975
6	-6.437255	-2.274262	0.132873
6	-7.192648	-2.150120	1.418483
7	0.575784	0.889291	-0.133518
6	1.944743	0.586374	-0.151472
7	2.714048	1.597526	0.264911
6	4.033194	1.444265	0.243272
6	4.680928	0.275972	-0.170531
6	3.862511	-0.763420	-0.613795
6	2.482559	-0.609097	-0.620355
7	6.098032	0.227540	-0.160154
6	6.674213	-0.964180	-0.780562
6	8.176870	-0.821548	-0.903642
7	8.779771	-0.599582	0.442714
6	8.171174	0.587511	1.110502
6	6.669041	0.418587	1.183086
8	-6.828028	-2.957211	-0.793562
1	-1.307936	-3.056988	0.081732
1	-7.120407	-1.141042	1.823981
1	-8.231895	-2.447487	1.285099
1	-6.710265	-2.828797	2.131893
1	-3.338349	-4.126111	-0.739461

1	-3.198844	-4.000593	1.013039
1	-4.792623	-4.199868	0.266804
1	0.368127	1.875304	-0.031089
1	4.616668	2.301696	0.562182
1	4.280753	-1.695705	-0.970711
1	1.845219	-1.399694	-0.982186
1	6.440588	-1.870232	-0.200302
1	6.268220	-1.087233	-1.784683
1	8.618173	-1.722556	-1.325006
1	8.447836	0.040148	-1.513857
1	9.800657	-0.445836	0.332013
1	8.600332	0.670371	2.107071
1	8.447733	1.458383	0.515969
1	6.425234	-0.443276	1.822777
1	6.253215	1.312089	1.644148
1	-5.428525	2.116940	1.528168
1	-3.871144	2.904558	1.827034
1	-4.481251	4.766352	0.336232
1	-5.967159	4.494900	1.256350
1	-6.177094	4.430249	-1.417708
1	-6.868011	3.067905	-0.528919
1	-4.249822	3.162819	-2.054012
1	-5.395372	1.829732	-1.955743
1	-3.063053	2.402364	-0.313814
1	8.629635	-1.430290	1.023433
8	11.405008	0.153986	-0.177439
1	11.948962	0.502458	0.538487
1	11.964491	-0.494826	-0.619838

Palbociclib-prot_O1

Atomic Number	Coordinates (Angstroms) X	Y	Z
6	-3.918165	3.039686	-1.190982
6	-3.071315	2.376797	-0.071164
6	-3.554633	2.961476	1.268746
6	-4.168169	4.302482	0.852976
6	-4.895970	3.968540	-0.454983
7	-3.061525	0.881988	-0.095204
6	-4.217342	0.194146	-0.058879
6	-4.270943	-1.240077	-0.083693
6	-3.090136	-1.944336	-0.297841
6	-1.878261	-1.209709	-0.290098
6	-1.857717	0.192661	-0.138096
6	-0.598259	-1.804744	-0.388783
7	0.522854	-1.135801	-0.309993
6	0.408029	0.203398	-0.126085
7	-0.740139	0.888713	-0.053401
8	-5.292617	0.913035	-0.003486
6	-3.033386	-3.415666	-0.572633
6	-5.602072	-1.850020	0.154420
6	-5.777787	-3.264651	0.607510

7	1.528045	0.943670	-0.019334
6	2.882184	0.544745	-0.040907
7	3.704490	1.480784	-0.514953
6	5.007699	1.224265	-0.547328
6	5.584536	0.020851	-0.113376
6	4.701043	-0.934976	0.398199
6	3.338410	-0.674631	0.441943
7	6.972489	-0.180863	-0.232047
6	7.450779	-1.496463	0.201010
6	8.924410	-1.659240	-0.120654
7	9.702328	-0.612929	0.545384
6	9.257895	0.690878	0.049075
6	7.789638	0.891399	0.367825
8	-6.611274	-1.141769	0.055200
1	-0.506778	-2.876199	-0.525361
1	-6.660790	-3.292384	1.246191
1	-5.968702	-3.900867	-0.261736
1	-4.913940	-3.645395	1.146035
1	-2.250092	-3.623815	-1.299119
1	-2.796950	-3.964467	0.343940
1	-3.967421	-3.787850	-0.978482
1	1.382417	1.947338	-0.024787
1	5.630230	2.010358	-0.958700
1	5.058953	-1.880755	0.781591
1	2.656130	-1.399048	0.858696
1	7.294640	-1.631018	1.282427
1	6.887248	-2.269236	-0.324025
1	9.254282	-2.637354	0.231320
1	9.055123	-1.628118	-1.211982
1	10.679069	-0.733655	0.295772
1	9.833322	1.480526	0.533681
1	9.400818	0.781146	-1.037469
1	7.644827	0.895432	1.458445
1	7.473306	1.856965	-0.020846
1	-4.319966	2.325699	1.716619
1	-2.731135	3.057425	1.977302
1	-3.373897	5.030474	0.659978
1	-4.825709	4.714321	1.619596
1	-5.142490	4.854411	-1.041577
1	-5.825206	3.439447	-0.231654
1	-3.242091	3.625545	-1.815913
1	-4.414358	2.315632	-1.835880
1	-2.032370	2.641709	-0.217346
1	-6.078388	0.273161	-0.020480
8	-9.192372	-2.427527	0.274797
1	-8.345946	-1.959536	0.215750
1	-8.946626	-3.329616	0.505551

Copanlisib-neut

Atomic Number	Coordinates (Angstroms) X	Y	Z

6	-7.630547	-1.619429	0.785523
7	-7.687088	-0.461207	-0.111898
6	-8.930540	0.266215	0.159996
6	-10.134761	-0.637585	-0.010060
8	-10.054873	-1.758625	0.865202
6	-8.859645	-2.491187	0.615353
6	-6.554938	0.436701	0.125334
6	-5.202179	-0.183973	-0.200847
6	-4.119875	0.872944	-0.128407
8	-2.863575	0.210950	-0.324477
6	-1.733421	0.946329	-0.235045
6	-0.537444	0.230261	-0.345896
6	0.699251	0.881876	-0.260787
6	0.709622	2.280228	-0.076130
6	-0.487308	2.986522	0.026758
6	-1.705616	2.334628	-0.046194
7	1.856505	0.116557	-0.350057
6	2.982034	0.744136	-0.260991
7	3.116338	2.077147	-0.070882
6	2.005807	2.934442	-0.028104
7	2.281405	4.182495	0.022672
6	3.749755	4.297156	0.085002
6	4.315395	2.910924	-0.263727
7	4.167006	0.004850	-0.414441
6	5.117307	-0.080379	0.583165
8	5.037102	0.576407	1.610899
8	-0.591098	-1.125584	-0.562348
6	-0.736644	-1.889954	0.645978
6	6.233792	-1.010929	0.331924
6	7.063232	-1.379357	1.393075
7	8.086155	-2.203036	1.258396
6	8.305510	-2.681205	0.013566
7	7.581588	-2.379782	-1.085129
6	6.562490	-1.555597	-0.909668
7	9.335699	-3.522000	-0.144364
1	5.998511	-1.306375	-1.803487
1	9.524723	-3.922531	-1.050098
1	9.892457	-3.790841	0.652137
1	6.874617	-0.989706	2.388130
1	4.053395	-0.807777	-1.010688
1	4.104157	5.062057	-0.603004
1	4.038435	4.595389	1.095237
1	5.122887	2.604393	0.394684
1	4.634138	2.837761	-1.305464
1	-0.798069	-2.934341	0.345265
1	0.132170	-1.736592	1.289076
1	-1.648995	-1.598366	1.170515
1	-0.460881	4.059554	0.169750
1	-2.624193	2.898059	0.040889
1	-4.116846	1.363298	0.850290
1	-4.254762	1.631560	-0.905179
1	-5.223559	-0.616768	-1.205503
1	-4.955348	-0.979113	0.506807

1	-6.553950	0.776465	1.175075
1	-6.704676	1.323104	-0.498363
1	-8.926618	0.664770	1.188595
1	-9.011898	1.110282	-0.528541
1	-11.050218	-0.102210	0.242015
1	-10.202666	-0.992618	-1.046096
1	-8.838485	-3.316609	1.326976
1	-8.894317	-2.899476	-0.402649
1	-7.561074	-1.287875	1.835856
1	-6.749642	-2.222439	0.560910
1	-3.324478	-1.397522	-2.232173
8	-2.578524	-1.980020	-2.411708
1	-1.897989	-1.672325	-1.790491

Copanlisib-prot_N1

Atomic Number	Coordinates (Angstroms) X	Y	Z
6	6.451550	-1.382177	-0.871244
6	6.145284	-0.860771	0.387154
6	7.048814	-1.163683	1.410267
7	8.118363	-1.911415	1.225534
6	8.311156	-2.377314	-0.030250
7	7.516570	-2.130348	-1.094863
6	4.988796	-0.011120	0.685412
8	4.894334	0.672803	1.685038
7	9.386264	-3.144119	-0.235625
7	3.958861	-0.041815	-0.265484
6	2.831604	0.738568	-0.159787
7	1.648155	0.143418	-0.239752
6	0.452904	0.864312	-0.175758
6	0.477817	2.258088	-0.052935
6	1.774864	2.913053	-0.056991
7	2.914288	2.053407	-0.040095
6	-0.728154	2.950373	0.019415
6	-1.936419	2.274418	-0.012731
6	-1.955500	0.878170	-0.127818
6	-0.749850	0.169953	-0.212967
7	2.051923	4.151760	-0.128447
6	3.510025	4.298294	-0.232635
6	4.123142	2.899580	-0.044870
8	-3.071162	0.125289	-0.153995
6	-4.339573	0.793755	-0.078566
6	-5.414597	-0.270832	-0.124559
6	-6.786094	0.377553	0.019481
7	-7.901153	-0.545964	-0.199679
6	-7.928561	-1.585018	0.834577
6	-9.145769	-2.474416	0.669919
8	-10.351963	-1.720073	0.728039
6	-10.350475	-0.713078	-0.279029
6	-9.156903	0.206862	-0.121321
8	-0.733940	-1.195373	-0.280620

6	-0.964547	-1.709401	-1.605879
1	5.826621	-1.175153	-1.735056
1	9.563557	-3.529720	-1.150510
1	9.998551	-3.368532	0.533770
1	6.879335	-0.784851	2.413123
1	3.834958	-0.907316	-0.786112
1	3.746220	4.715941	-1.211553
1	3.871162	4.992154	0.523749
1	4.633833	2.800152	0.909257
1	4.785074	2.593797	-0.854190
1	-0.948135	-2.794112	-1.520676
1	-1.936403	-1.378576	-1.975775
1	-0.171477	-1.373645	-2.278056
1	-0.717534	4.029017	0.108805
1	-2.860174	2.831560	0.055945
1	-4.388498	1.362500	0.854918
1	-4.429626	1.484340	-0.922256
1	-5.350626	-0.808303	-1.074766
1	-5.231555	-0.983229	0.683192
1	-6.866978	0.838104	1.018903
1	-6.881339	1.183929	-0.713816
1	-9.233638	0.727752	0.848008
1	-9.174792	0.960639	-0.911713
1	-11.279046	-0.152778	-0.169497
1	-10.336364	-1.190517	-1.266920
1	-9.190125	-3.206199	1.476591
1	-9.098765	-3.004648	-0.289742
1	-7.945058	-1.126574	1.838281
1	-7.036556	-2.208649	0.760949
1	1.624448	-0.894263	-0.279045
8	1.867720	-2.645758	-0.382928
1	1.072284	-3.043227	-0.007833
1	2.590744	-2.960170	0.173319

Copanlisib-prot_N2

Atomic Number	Atomic Type	Coordinates (Angstroms) X	Y	Z
6	0	6.494254	-1.849198	-0.887582
6	0	6.142499	-1.301278	0.346502
6	0	6.984275	-1.619978	1.414856
7	0	8.038291	-2.404199	1.292727
6	0	8.277760	-2.891949	0.054603
7	0	7.544711	-2.634631	-1.049872
6	0	4.994158	-0.411721	0.577639
8	0	4.879671	0.270923	1.583257
7	0	9.338826	-3.694279	-0.089737
7	0	4.037540	-0.383880	-0.425949
6	0	2.845961	0.330650	-0.276835
7	0	1.721468	-0.292026	-0.313546
6	0	0.563597	0.458862	-0.228584
6	0	0.573896	1.866270	-0.086225

6	0	1.859593	2.479718	-0.083553
7	0	2.963599	1.689335	-0.150453
6	0	-0.622292	2.587996	0.008916
6	0	-1.829889	1.929149	-0.024828
6	0	-1.860247	0.527574	-0.160234
6	0	-0.670992	-0.201103	-0.264224
7	0	2.188967	3.747863	-0.065618
6	0	3.647120	3.922082	-0.004034
6	0	4.169042	2.526444	-0.367239
8	0	-2.989719	-0.199045	-0.190721
6	0	-4.249365	0.484647	-0.091798
6	0	-5.336191	-0.567574	-0.143166
6	0	-6.699582	0.092925	0.020589
7	0	-7.825563	-0.818736	-0.192398
6	0	-7.854124	-1.861687	0.837979
6	0	-9.081271	-2.738623	0.680521
8	0	-10.279570	-1.972842	0.752053
6	0	-10.276880	-0.962043	-0.251161
6	0	-9.073238	-0.054155	-0.100062
8	0	-0.724386	-1.564363	-0.358792
6	0	-0.931057	-2.028660	-1.702504
1	0	5.921026	-1.635409	-1.784803
1	0	9.544634	-4.100445	-0.989420
1	0	9.903412	-3.931487	0.711401
1	0	6.778815	-1.222949	2.403716
1	0	3.933786	-1.222052	-0.989298
1	0	3.972610	4.679159	-0.712159
1	0	3.936631	4.213898	1.005457
1	0	4.982994	2.210351	0.276309
1	0	4.454464	2.446066	-1.416136
1	0	-0.973498	-3.114735	-1.650414
1	0	-1.870472	-1.638774	-2.099644
1	0	-0.098577	-1.718331	-2.337855
1	0	-0.602684	3.665968	0.114377
1	0	-2.749225	2.491834	0.055790
1	0	-4.281981	1.038493	0.851062
1	0	-4.339712	1.188659	-0.924079
1	0	-5.285431	-1.092977	-1.100844
1	0	-5.153881	-1.292395	0.653555
1	0	-6.765103	0.547353	1.023957
1	0	-6.795131	0.905081	-0.706244
1	0	-9.136552	0.463472	0.871987
1	0	-9.090779	0.702920	-0.887299
1	0	-11.199136	-0.393388	-0.131806
1	0	-10.275444	-1.435783	-1.240935
1	0	-9.125697	-3.473094	1.484740
1	0	-9.047827	-3.265496	-0.281546
1	0	-7.857575	-1.407062	1.843535
1	0	-6.968991	-2.493732	0.754298
1	0	1.521267	4.523295	0.034941
8	0	0.582221	6.083658	0.140599
1	0	0.841048	6.634933	0.888206
1	0	-0.375769	6.003754	0.214424

Copanlisib-prot_N3

Atomic Number	Coordinates (Angstroms) X	Y	Z
6	8.774732	0.383797	-0.215294
7	7.475804	-0.354783	-0.160585
6	7.541537	-1.565394	-1.036621
6	8.746742	-2.405862	-0.658215
8	9.950282	-1.659660	-0.755359
6	9.913445	-0.553276	0.133934
6	6.334356	0.547638	-0.491915
6	4.991915	-0.151453	-0.340232
6	3.891210	0.889902	-0.316675
8	2.650212	0.183977	-0.254230
6	1.511240	0.911827	-0.159682
6	1.468807	2.312011	-0.156391
6	0.247907	2.954895	-0.051645
6	-0.936422	2.227250	0.043361
6	-0.910095	0.816917	0.037853
6	0.329167	0.170770	-0.066072
6	-2.232353	2.868110	0.188863
7	-3.335536	2.000873	0.213242
6	-3.185198	0.655943	0.215122
7	-2.057152	0.034609	0.120017
6	-4.512274	2.789242	0.617729
6	-3.979071	4.214821	0.402812
7	-2.510706	4.109107	0.325624
7	-4.352712	-0.109243	0.376162
6	-5.386235	-0.077832	-0.537589
8	-5.394728	0.702172	-1.478819
8	0.397430	-1.199483	-0.107038
6	0.632551	-1.785007	1.181150
6	-6.478249	-1.041231	-0.305294
6	-6.713761	-1.710744	0.895480
7	-7.717514	-2.555499	1.061557
6	-8.520081	-2.750765	-0.006100
7	-8.391620	-2.150059	-1.210004
6	-7.382486	-1.308065	-1.335088
7	-9.534567	-3.612399	0.140264
1	-6.084425	-1.547271	1.765170
1	-9.674854	-4.081731	1.021438
1	-10.161104	-3.786945	-0.630176
1	-7.267378	-0.818698	-2.296790
1	-4.189383	-0.988515	0.854499
1	-4.273530	4.881575	1.210710
1	-4.352951	4.636235	-0.532853
1	-5.375860	2.561353	-0.000180
1	-4.736737	2.579499	1.665522
1	0.682301	-2.861748	1.029419
1	1.576835	-1.425083	1.596154
1	-0.188325	-1.543498	1.860307

1	0.210021	4.037011	-0.046235
1	2.376677	2.893306	-0.236944
1	3.915645	1.507769	-1.220040
1	3.988950	1.536648	0.559903
1	4.970232	-0.721715	0.593971
1	4.814371	-0.839732	-1.168616
1	6.493814	0.905472	-1.510657
1	6.412120	1.393000	0.195079
1	8.879247	0.769707	-1.230833
1	8.718939	1.210473	0.492656
1	10.858990	-0.021919	0.038796
1	9.806192	-0.913748	1.164917
1	8.824435	-3.247577	-1.344620
1	8.633860	-2.786971	0.364690
1	7.606280	-1.210804	-2.067166
1	6.628009	-2.141393	-0.895655
1	7.338074	-0.693014	0.816252
8	7.103512	-1.424601	2.427831
1	6.247179	-1.866182	2.478556
1	7.752426	-2.133945	2.512777

Copanlisib-prot_N4

Atomic Number	Coordinates (Angstroms) X	Y	Z
6	6.277592	-0.777481	-1.031057
6	5.880576	-0.413497	0.248329
6	6.689841	-0.825619	1.302043
7	7.786715	-1.554084	1.102924
6	8.044541	-1.839308	-0.158458
7	7.378774	-1.504330	-1.242045
6	4.679933	0.419815	0.552381
8	4.612869	1.073277	1.577374
7	9.217196	-2.668745	-0.370819
7	3.689382	0.394964	-0.392333
6	2.451807	1.043970	-0.210232
7	1.380689	0.327129	-0.247310
6	0.167830	1.000644	-0.131996
6	0.076355	2.398873	0.030120
6	1.317397	3.153978	0.024636
7	2.491122	2.386021	-0.044610
6	-1.167998	3.013377	0.151163
6	-2.333150	2.267722	0.119847
6	-2.258968	0.878172	-0.043954
6	-1.014539	0.250609	-0.167993
7	1.495028	4.420305	0.046122
6	2.950474	4.651646	0.053973
6	3.611990	3.308996	-0.295754
8	-3.333840	0.057855	-0.088175
6	-4.636156	0.649856	-0.001715
6	-5.650660	-0.469687	-0.102190
6	-7.056438	0.092472	0.070623

7	-8.118019	-0.871278	-0.226703
6	-8.087505	-1.989990	0.720489
6	-9.252042	-2.930503	0.477356
8	-10.498983	-2.252111	0.588607
6	-10.553258	-1.169236	-0.335133
6	-9.414224	-0.199057	-0.096332
8	-0.966528	-1.114831	-0.296320
6	-1.105260	-1.552158	-1.655377
1	5.726485	-0.475007	-1.914523
1	9.499517	-2.657011	-1.355092
1	10.000287	-2.345896	0.206976
1	6.443390	-0.567766	2.325300
1	3.644140	-0.413785	-1.004940
1	3.218801	5.430428	-0.656955
1	3.249792	4.988291	1.048885
1	4.465156	3.081592	0.336625
1	3.897251	3.242849	-1.347566
1	-1.073653	-2.640079	-1.637410
1	-2.059165	-1.216199	-2.068263
1	-0.281626	-1.166743	-2.260906
1	-1.221795	4.087659	0.275029
1	-3.289311	2.761196	0.223743
1	-4.729603	1.178472	0.952283
1	-4.759989	1.367778	-0.818212
1	-5.556912	-0.956187	-1.077172
1	-5.428131	-1.209895	0.669950
1	-7.169812	0.475653	1.099080
1	-7.190017	0.942893	-0.604736
1	-9.522391	0.236582	0.911335
1	-9.473288	0.613976	-0.823485
1	-11.512526	-0.673134	-0.187247
1	-10.509675	-1.564754	-1.357738
1	-9.256343	-3.726627	1.221902
1	-9.173549	-3.377612	-0.521752
1	-8.131741	-1.615731	1.757775
1	-7.160881	-2.553383	0.602132
1	8.985246	-3.676516	-0.084686
8	8.432917	-5.106492	0.388083
1	8.830439	-5.845480	-0.088618
1	8.613399	-5.278586	1.320512

Copanlisib-prot_N5

Atomic Number	Coordinates (Angstroms) X	Y	Z
6	9.422547	-0.089551	0.287745
7	8.137892	-0.794954	0.261669
6	8.187155	-1.792944	-0.811102
6	9.358297	-2.735779	-0.612020
8	10.593102	-2.028037	-0.568516
6	10.569156	-1.061635	0.477511
6	7.070625	0.178027	0.020837

6	5.672735	-0.428723	0.039117
6	4.634978	0.673814	0.058935
8	3.346526	0.049811	-0.007645
6	2.251463	0.841765	0.051100
6	2.291828	2.239838	0.137237
6	1.107718	2.953722	0.191672
6	-0.122263	2.300156	0.158802
6	-0.179298	0.893339	0.073644
6	1.021972	0.175031	0.016886
6	-1.383826	3.014960	0.248216
7	-2.537113	2.219061	0.154867
6	-2.465361	0.869534	0.093398
7	-1.375162	0.182673	0.035974
6	-3.689480	3.061554	0.519757
6	-3.057165	4.458500	0.412732
7	-1.596325	4.263179	0.428415
7	-3.686090	0.169299	0.139604
6	-4.662404	0.327146	-0.812268
8	-4.585107	1.137917	-1.717863
8	1.008473	-1.192366	-0.101521
6	1.113446	-1.862709	1.162010
6	-5.847869	-0.555856	-0.671858
6	-6.213065	-1.182182	0.490428
7	-7.321888	-1.937218	0.505284
6	-8.092666	-2.096642	-0.604774
7	-7.776981	-1.493651	-1.766422
6	-6.696188	-0.747859	-1.782939
7	-9.166207	-2.856056	-0.520614
1	-5.679516	-1.109041	1.429841
1	-9.743856	-2.976739	-1.341575
1	-6.453147	-0.262500	-2.722358
1	-3.613740	-0.739158	0.587412
1	-3.366508	5.104594	1.231742
1	-3.338755	4.945525	-0.523414
1	-4.519969	2.920196	-0.165685
1	-3.998916	2.818253	1.538167
1	1.110115	-2.930336	0.949818
1	2.044445	-1.587758	1.663223
1	0.260857	-1.607501	1.795645
1	1.134878	4.034067	0.258976
1	3.236478	2.764839	0.159228
1	4.755024	1.345078	-0.797546
1	4.704727	1.259113	0.980574
1	5.547163	-1.056049	0.926160
1	5.504576	-1.050597	-0.843360
1	7.232379	0.687864	-0.944161
1	7.143188	0.940639	0.802184
1	9.576856	0.464715	-0.653439
1	9.420554	0.631922	1.107973
1	11.523113	-0.535232	0.445721
1	10.477360	-1.576323	1.442289
1	9.423810	-3.437554	-1.443504
1	9.233757	-3.299830	0.321095

1	8.280483	-1.297115	-1.792634
1	7.269016	-2.382272	-0.814226
1	-9.408439	-3.310082	0.355594
1	-7.632300	-2.412937	1.371054
8	-8.743634	-3.455578	2.320186
1	-8.367845	-4.266837	2.681923
1	-9.211578	-3.040296	3.054324

Copanlisib-prot_O6

Atomic Number	Coordinates (Angstroms) X	Y	Z
6	6.834763	-1.571339	1.277347
6	6.080302	-1.072162	0.206289
6	6.645701	-1.215726	-1.066862
7	7.805728	-1.803359	-1.265215
6	8.438806	-2.274335	-0.163762
7	7.993697	-2.170033	1.112135
6	4.812362	-0.424454	0.451945
8	4.590838	0.016358	1.632673
7	9.604124	-2.889816	-0.350625
7	3.924288	-0.291363	-0.529926
6	2.723253	0.467555	-0.421095
7	2.894053	1.798365	-0.278646
6	1.800238	2.670490	-0.154584
6	0.494908	2.028735	-0.169388
6	0.454675	0.629709	-0.353203
7	1.602694	-0.153591	-0.478227
6	-0.788532	-0.011971	-0.388757
6	-1.970716	0.724799	-0.246029
6	-1.916026	2.112896	-0.068015
6	-0.688066	2.750723	-0.032121
6	4.122191	2.605504	-0.394839
6	3.570242	4.009215	-0.071384
7	2.100037	3.908885	-0.063155
8	-3.113228	0.004883	-0.283598
6	-4.358099	0.704150	-0.153708
6	-5.462882	-0.328997	-0.221291
6	-6.816343	0.346910	-0.041409
7	-7.955335	-0.550802	-0.246029
6	-9.192847	0.229954	-0.152199
6	-10.408470	-0.663290	-0.294839
8	-10.420539	-1.669606	0.712839
6	-9.232203	-2.450739	0.640431
6	-7.993595	-1.588616	0.789271
8	-0.863188	-1.373572	-0.525106
6	-1.082046	-1.790782	-1.880824
1	6.152676	-0.817813	-1.948951
1	10.117080	-3.249372	0.440651
1	6.463632	-1.485986	2.293125
1	4.038668	-0.835276	-1.381405
1	3.893051	4.742718	-0.807789

1	3.908764	4.348832	0.908464
1	4.879261	2.280629	0.316628
1	4.510960	2.525249	-1.410529
1	-1.144633	-2.877234	-1.864755
1	-2.015155	-1.371392	-2.263116
1	-0.246180	-1.475671	-2.509686
1	-0.644300	3.823721	0.105745
1	-2.823317	2.689946	0.044952
1	-4.374862	1.234752	0.803321
1	-4.447486	1.432729	-0.965354
1	-5.423171	-0.836913	-1.188956
1	-5.289158	-1.072115	0.560428
1	-6.867171	0.799109	0.963858
1	-6.909083	1.162307	-0.764890
1	-9.245814	0.752699	0.817740
1	-9.203994	0.983603	-0.942816
1	-11.323083	-0.082535	-0.174967
1	-10.416384	-1.141433	-1.282474
1	-9.282822	-3.180852	1.448258
1	-9.209095	-2.982478	-0.319260
1	-7.988006	-1.129156	1.792613
1	-7.116918	-2.232294	0.705983
1	9.982211	-2.984171	-1.281503
1	3.601295	0.234758	1.952274
8	2.376278	0.443435	2.618720
1	1.968956	1.310294	2.484140
1	1.702075	-0.206003	2.376039

Olaparib-neut

Atomic Number	Coordinates (Angstroms) X	Y	Z
6	-3.076963	2.102141	-2.585603
6	-3.360558	0.938154	-1.896453
6	-3.769111	1.001302	-0.553223
6	-3.886868	2.257197	0.062034
6	-3.597827	3.431346	-0.642031
6	-3.191525	3.351569	-1.959732
6	-4.077228	-0.185923	0.229681
7	-4.470635	-0.114169	1.457317
7	-4.591616	1.113055	2.018555
6	-4.332874	2.324498	1.453966
6	-3.929885	-1.561653	-0.358582
6	-2.489140	-1.928424	-0.657602
6	-1.461651	-1.549449	0.201983
6	-0.135345	-1.886517	-0.066544
6	0.130288	-2.638206	-1.200328
6	-0.863261	-3.045765	-2.068771
6	-2.174656	-2.678019	-1.792653
6	0.920299	-1.525891	0.938024
8	0.767593	-1.912346	2.108028
9	1.408293	-2.985882	-1.464050

8	-4.480297	3.366734	2.098892
7	1.970594	-0.793403	0.547350
6	2.136693	-0.173447	-0.771364
6	3.493490	-0.551626	-1.354742
7	4.557720	-0.162876	-0.425551
6	4.399282	-0.839928	0.865836
6	3.051907	-0.481740	1.486160
6	5.059006	1.102778	-0.525558
8	4.759078	1.840142	-1.470528
6	6.001076	1.562415	0.532117
6	6.731358	2.857985	0.308304
6	5.591027	2.823925	1.269387
1	-1.678033	-0.977564	1.099178
1	-0.605450	-3.633884	-2.940859
1	-2.965407	-2.978060	-2.471592
1	3.024260	0.590512	1.709585
1	2.898660	-1.041283	2.404840
1	5.195005	-0.571977	1.553678
1	4.451380	-1.915764	0.686936
1	3.641491	-0.056584	-2.310204
1	3.552414	-1.631623	-1.498601
1	2.092158	0.912618	-0.639082
1	1.330579	-0.466901	-1.437998
1	7.733345	2.912068	0.712034
1	6.574601	3.350406	-0.641579
1	5.799776	2.857738	2.330106
1	4.659066	3.277280	0.955384
1	-4.906630	1.105334	2.982706
1	-4.515037	-1.631734	-1.279002
1	-4.349992	-2.275758	0.352879
1	-3.698678	4.387687	-0.143957
1	-2.963914	4.254366	-2.513320
1	-2.763675	2.048099	-3.621314
1	-3.270138	-0.018848	-2.395454
1	6.514634	0.809221	1.109296
1	-0.584106	-3.111935	2.440010
8	-1.310391	-3.740445	2.605332
1	-0.996447	-4.291759	3.329466

Olaparib-prot_N1

Atomic Number	Coordinates (Angstroms) X	Y	Z
6	-3.420850	-0.723697	-1.423138
7	-2.262292	-0.960234	-0.558395
6	-2.285109	-0.182048	0.684088
6	-3.588383	-0.437259	1.432631
7	-4.726766	-0.120852	0.566061
6	-4.712061	-0.951777	-0.642540
6	-1.265246	-1.762986	-0.964567
8	-1.230549	-2.285810	-2.084667
6	-5.182201	1.165673	0.556798

6	-6.210438	1.530065	-0.457426
6	-5.831839	2.660554	-1.396224
6	-6.865746	2.878373	-0.342792
6	-0.125277	-2.029693	-0.021173
6	1.175013	-1.752428	-0.435991
6	2.264833	-2.044326	0.380725
6	2.046886	-2.653640	1.615803
6	0.759413	-2.963260	2.038391
6	-0.298621	-2.638089	1.213087
6	3.672904	-1.720986	-0.086687
6	3.824671	-0.255323	-0.367044
6	3.879637	0.764882	0.657174
6	3.948338	2.114501	0.264980
6	3.984495	2.469287	-1.160968
7	3.952452	1.397798	-2.010627
7	3.870432	0.109864	-1.608797
6	3.998120	3.126731	1.221719
6	3.980524	2.793893	2.565552
6	3.918627	1.453865	2.962586
6	3.868254	0.441749	2.021317
8	4.045576	3.607123	-1.603089
9	-1.551821	-2.926515	1.620524
8	-4.772441	1.998962	1.371904
1	1.322230	-1.301065	-1.412784
1	0.570397	-3.446500	2.988908
1	2.890600	-2.892457	2.253831
1	-3.387925	0.311131	-1.782131
1	-3.372661	-1.397266	-2.274609
1	-5.562201	-0.735131	-1.281737
1	-4.777779	-1.996057	-0.330564
1	-3.630078	0.171860	2.331066
1	-3.661337	-1.489843	1.710760
1	-2.225923	0.879018	0.419083
1	-1.427856	-0.422247	1.306971
1	-7.901022	2.929662	-0.651748
1	-6.594191	3.483582	0.511241
1	-6.146860	2.564703	-2.426495
1	-4.856692	3.104531	-1.238658
1	3.982201	1.539979	-3.017332
1	4.397740	-2.012468	0.674792
1	3.901696	-2.278314	-0.997328
1	4.052836	4.159068	0.900029
1	4.019640	3.575386	3.314470
1	3.912115	1.204776	4.016388
1	3.827841	-0.591407	2.341523
1	-6.806676	0.732996	-0.874238
1	3.832745	-0.602257	-2.416483
8	3.740063	-1.567322	-3.632412
1	3.889594	-2.484950	-3.370747
1	4.404304	-1.382125	-4.308306

Olaparib-prot_N2

Atomic Number	Coordinates (Angstroms) X	Y	Z
6	2.102477	1.384763	-1.567842
7	0.721862	1.169793	-1.004123
6	0.801830	0.413314	0.308988
6	1.743293	1.116832	1.266497
7	3.078595	1.207273	0.678242
6	3.044646	2.010698	-0.547033
6	-0.089438	2.440566	-0.940138
8	0.429844	3.455033	-1.288228
6	3.885226	0.104581	0.797073
6	5.163387	0.101409	0.037563
6	5.342468	-1.054889	-0.930364
6	6.146832	-0.999675	0.323985
6	-1.464035	2.297175	-0.425130
6	-2.180029	1.098201	-0.523246
6	-3.432415	0.948911	0.052696
6	-3.997060	2.046483	0.709781
6	-3.328926	3.259958	0.790223
6	-2.071061	3.369073	0.228090
6	-4.157857	-0.384957	0.025390
6	-3.216605	-1.536580	-0.198148
6	-2.152521	-1.827330	0.753851
6	-1.174574	-2.766438	0.392398
6	-1.328899	-3.497491	-0.866937
7	-2.448372	-3.170371	-1.572529
7	-3.342234	-2.186985	-1.305870
6	-0.074185	-3.012973	1.221601
6	0.040147	-2.333820	2.419647
6	-0.954908	-1.425599	2.810153
6	-2.038811	-1.169095	1.990718
8	-0.543027	-4.349548	-1.288910
9	-1.414250	4.528057	0.360522
8	3.564348	-0.840759	1.520030
1	-1.738185	0.260404	-1.049971
1	-3.755548	4.113208	1.302458
1	-4.969653	1.944921	1.178875
1	2.452715	0.388994	-1.847221
1	2.008839	2.000707	-2.459122
1	4.027193	2.085115	-1.002586
1	2.720965	3.016736	-0.281642
1	1.776284	0.561806	2.200375
1	1.393465	2.130663	1.478052
1	1.171684	-0.580162	0.047083
1	-0.197181	0.329227	0.725435
1	7.191712	-0.731877	0.244907
1	5.891068	-1.688594	1.117384
1	5.825265	-0.829447	-1.871563
1	4.530391	-1.770210	-0.973333
1	-2.596993	-3.645051	-2.456742
1	-4.688784	-0.510999	0.973122
1	-4.903773	-0.392325	-0.771563

1	0.673018	-3.733770	0.912848
1	0.892479	-2.510498	3.064623
1	-0.868203	-0.911028	3.759738
1	-2.791554	-0.453548	2.299485
1	5.569780	1.055325	-0.262673
1	0.258353	0.498927	-1.673121
8	0.006933	-0.912102	-2.599323
1	0.380780	-1.709979	-2.204249
1	-0.913496	-1.128719	-2.792783

Olaparib-prot_N3

Atomic Number	Coordinates (Angstroms) X	Y	Z
6	-2.757199	-0.014652	-1.696433
7	-1.684538	-0.586678	-0.888980
6	-1.760527	-0.198020	0.516847
6	-3.104253	-0.592915	1.110025
7	-4.250268	-0.066172	0.299515
6	-4.102306	-0.454924	-1.153518
6	-0.709858	-1.319022	-1.462453
8	-0.634209	-1.491143	-2.681283
6	-4.516349	1.399560	0.484172
6	-5.744593	1.861788	-0.155818
6	-5.609874	3.154673	-0.959252
6	-6.314517	3.174301	0.341311
6	0.338363	-1.932694	-0.579176
6	1.680105	-1.644384	-0.823687
6	2.692191	-2.239489	-0.074272
6	2.343826	-3.157307	0.918119
6	1.013786	-3.477390	1.164851
6	0.038437	-2.852777	0.413296
6	4.148897	-1.913819	-0.345283
6	4.387239	-0.442517	-0.548220
6	4.231879	0.501665	0.548317
6	4.424218	1.867114	0.286488
6	4.787266	2.284583	-1.068169
7	4.906119	1.260355	-1.956976
7	4.712491	-0.062783	-1.738628
6	4.286429	2.819426	1.302304
6	3.957409	2.408760	2.579391
6	3.769441	1.046678	2.852934
6	3.903456	0.100267	1.854506
8	4.983051	3.451892	-1.418471
9	-1.258130	-3.149690	0.651820
8	-3.736941	2.050243	1.115815
1	1.922441	-0.936683	-1.610285
1	0.728846	-4.192859	1.926179
1	3.122511	-3.627627	1.508653
1	-2.673351	1.077725	-1.683422
1	-2.665357	-0.358199	-2.723097
1	-4.917563	-0.004652	-1.715872

1	-4.201947	-1.540501	-1.178223
1	-3.206738	-0.210672	2.123413
1	-3.225974	-1.676177	1.112161
1	-1.619976	0.882728	0.580200
1	-0.973035	-0.670601	1.097567
1	-7.394245	3.230739	0.341139
1	-5.808517	3.618980	1.188163
1	-6.190187	3.190559	-1.870727
1	-4.612846	3.573685	-1.004845
1	5.159090	1.496356	-2.910578
1	4.758500	-2.275608	0.486177
1	4.479947	-2.432621	-1.247113
1	4.442448	3.866393	1.073440
1	3.848007	3.137865	3.372948
1	3.518177	0.731801	3.858692
1	3.760147	-0.948060	2.085192
1	-6.422940	1.105301	-0.524129
1	-5.123125	-0.575050	0.625439
8	-6.426876	-1.545621	0.923186
1	-6.190727	-2.477990	0.842101
1	-6.758033	-1.447077	1.824277

Olaparib-prot_O4

Atomic Number	Coordinates (Angstroms) X	Y	Z
6	-2.817605	-0.867353	-1.823139
7	-1.720234	-1.138381	-0.895643
6	-1.818580	-0.407944	0.367494
6	-3.165182	-0.695177	1.017865
7	-4.236507	-0.316907	0.082746
6	-4.164856	-1.118775	-1.151824
6	-0.682868	-1.909616	-1.275474
8	-0.575723	-2.375744	-2.412622
6	-4.930551	0.793565	0.192798
6	-6.019654	1.131551	-0.724975
6	-6.011669	2.543897	-1.295187
6	-7.029482	2.170480	-0.283234
6	0.394931	-2.202571	-0.270970
6	1.713362	-1.865557	-0.570873
6	2.751491	-2.172818	0.305957
6	2.455930	-2.856077	1.486394
6	1.150911	-3.222236	1.796227
6	0.146642	-2.879537	0.913107
6	4.179350	-1.780432	-0.022708
6	4.288987	-0.361660	-0.511587
6	4.011442	0.760409	0.372804
6	4.079954	2.059521	-0.153794
6	4.446865	2.235653	-1.559241
7	4.693687	1.073267	-2.223520
7	4.616739	-0.193625	-1.749047
6	3.818367	3.174725	0.649749

6	3.490805	2.993208	1.979371
6	3.428097	1.700098	2.517433
6	3.683760	0.593843	1.729210
8	4.542251	3.324406	-2.133120
9	-1.127977	-3.212062	1.213123
8	-4.723746	1.653652	1.142773
1	1.917564	-1.345718	-1.501806
1	0.906820	-3.756809	2.705946
1	3.255829	-3.101131	2.176737
1	-2.763022	0.176768	-2.148122
1	-2.721929	-1.515178	-2.690191
1	-4.961723	-0.856642	-1.838135
1	-4.266538	-2.166865	-0.868565
1	-3.272201	-0.173466	1.964147
1	-3.275498	-1.764486	1.203519
1	-1.741815	0.663621	0.152748
1	-1.011514	-0.679269	1.042202
1	-8.040358	1.984367	-0.618910
1	-6.924391	2.566774	0.716544
1	-6.306833	2.626792	-2.331934
1	-5.194854	3.178916	-0.978058
1	4.957241	1.140859	-3.200683
1	4.802047	-1.914656	0.865053
1	4.572421	-2.433403	-0.804320
1	3.879593	4.166146	0.218157
1	3.286331	3.849798	2.610035
1	3.178612	1.566100	3.563231
1	3.635682	-0.397671	2.161679
1	-6.385404	0.345281	-1.363733
1	-3.831090	1.673747	1.627646
8	-2.610492	2.048325	2.515354
1	-2.719566	2.948387	2.848021
1	-1.760396	2.047519	2.057440

Olaparib-prot_O5

Atomic Number	Coordinates (Angstroms) X	Y	Z
6	-3.038256	-0.444359	-1.477304
7	-1.944101	-0.755562	-0.537638
6	-2.108683	-0.134156	0.789864
6	-3.461369	-0.526764	1.372110
7	-4.530678	-0.143521	0.449645
6	-4.374285	-0.817579	-0.840827
6	-0.930445	-1.499071	-0.883836
8	-0.833114	-1.892380	-2.111172
6	-5.025570	1.129164	0.546263
6	-5.972393	1.584658	-0.507029
6	-5.558451	2.837166	-1.258574
6	-6.690610	2.887981	-0.289266
6	0.137078	-1.887730	0.070139
6	1.462067	-1.547686	-0.206709

6	2.487467	-1.938669	0.648373
6	2.169514	-2.695915	1.778083
6	0.858696	-3.060428	2.060046
6	-0.137183	-2.644331	1.200544
6	3.928717	-1.571281	0.354651
6	4.077943	-0.194683	-0.231239
6	3.769045	0.991362	0.552790
6	3.890253	2.247871	-0.060665
6	4.340304	2.316367	-1.451277
7	4.598680	1.104923	-2.017133
7	4.474304	-0.122318	-1.457816
6	3.600618	3.421412	0.644009
6	3.189999	3.340422	1.960352
6	3.071656	2.090421	2.584245
6	3.355982	0.926903	1.894538
8	4.490757	3.358783	-2.094563
9	-1.414919	-2.978372	1.459006
8	-4.711246	1.866731	1.483469
1	1.675863	-0.963636	-1.096584
1	0.602746	-3.651174	2.930648
1	2.961054	-3.003530	2.452596
1	-3.002738	0.630079	-1.677849
1	-2.888746	-0.992581	-2.402017
1	-5.172780	-0.556245	-1.527893
1	-4.414062	-1.894315	-0.666395
1	-3.601912	-0.032401	2.328832
1	-3.511452	-1.606586	1.517642
1	-2.071870	0.947566	0.637072
1	-1.291628	-0.420366	1.445607
1	-7.695369	2.945474	-0.685457
1	-6.522842	3.386742	0.655447
1	-5.775164	2.862153	-2.317907
1	-4.620522	3.285863	-0.955898
1	4.917056	1.098169	-2.980224
1	4.509419	-1.643178	1.277565
1	4.350588	-2.285444	-0.355525
1	3.704486	4.378285	0.147589
1	2.962052	4.242813	2.514447
1	2.755139	2.035469	3.618912
1	3.263244	-0.030302	2.392825
1	-6.494991	0.829134	-1.073057
1	-0.065256	-2.569641	-2.273673
8	1.013574	-3.514533	-2.597368
1	0.718685	-4.193254	-3.218717
1	1.323510	-3.993656	-1.817076
